# Kraft Lignin-Derived Microporous Nitrogen-Doped Carbon Adsorbent for Air and Water Purification

**DOI:** 10.1021/acsami.3c15659

**Published:** 2024-01-09

**Authors:** Oleg Tkachenko, Alina Nikolaichuk, Nataliia Fihurka, Andreas Backhaus, Julie B. Zimmerman, Maria Strømme, Tetyana M. Budnyak

**Affiliations:** † Division of Nanotechnology and Functional Materials, Department of Materials Science and Engineering, The Ångström Laboratory, Uppsala University, Lägerhyddsvägen 1, Uppsala 751 03, Sweden; ‡ Center for Green Chemistry and Green Engineering, School of the Environment, Yale University, 195 Prospect Street, New Haven, Connecticut 06511, United States; § Department of Earth Sciences, Uppsala University, P.O. Box 256, Uppsala 751 05, Sweden

**Keywords:** lignin, microporous carbon, green chemistry, CO_2_ capture, herbicide removal, air and water purification

## Abstract

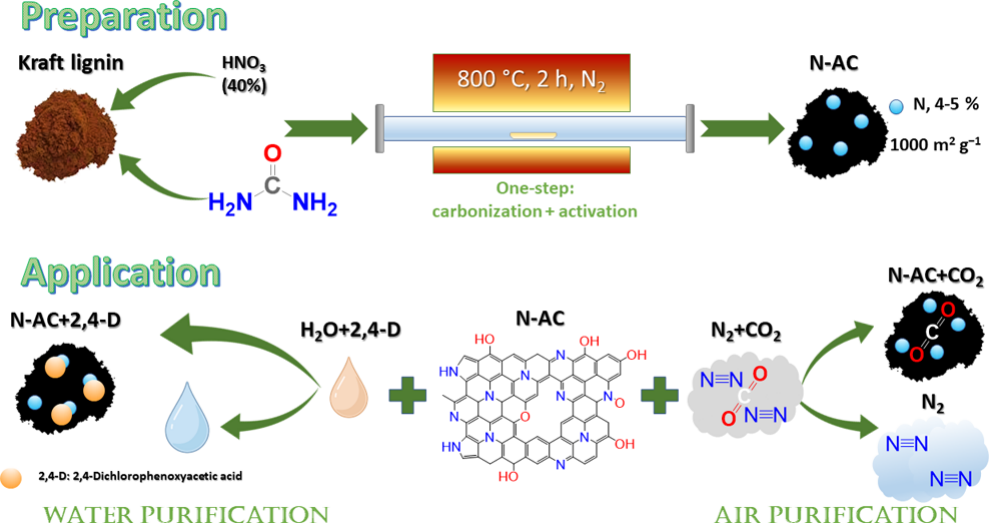

The study presents a streamlined one-step process for producing highly porous, metal-free, N-doped activated carbon (N-AC) for CO_2_ capture and herbicide removal from simulated industrially polluted and real environmental systems. N-AC was prepared from kraft lignin—a carbon-rich and abundant byproduct of the pulp industry, using nitric acid as the activator and urea as the N-dopant. The reported carbonization process under a nitrogen atmosphere renders a product with a high yield of 30% even at high temperatures up to 800 °C. N-AC exhibited a substantial high N content (4–5%), the presence of aliphatic and phenolic OH groups, and a notable absence of carboxylic groups, as confirmed by Fourier transform infrared spectroscopy, X-ray photoelectron spectroscopy, and Boehm’s titration. Porosity analysis indicated that micropores constituted the majority of the pore structure, with 86% of pores having diameters less than 0.6 nm. According to BET adsorption analysis, the developed porous structure of N-AC boasted a substantial specific surface area of 1000 m^2^ g^–1^. N-AC proved to be a promising adsorbent for air and water purification. Specifically, N-AC exhibited a strong affinity for CO_2_, with an adsorption capacity of 1.4 mmol g^–1^ at 0.15 bar and 20 °C, and it demonstrated the highest selectivity over N_2_ from the simulated flue gas system (27.3 mmol g^–1^ for 15:85 v/v CO_2_/N_2_ at 20 °C) among all previously reported nitrogen-doped AC materials from kraft lignin. Moreover, N-AC displayed excellent reusability and efficient CO_2_ release, maintaining an adsorption capacity of 3.1 mmol g^–1^ (at 1 bar and 25 °C) over 10 consecutive adsorption–desorption cycles, confirming N-AC as a useful material for CO_2_ storage and utilization. The unique cationic nature of N-AC enhanced the adsorption of herbicides in neutral and weakly basic environments, which is relevant for real waters. It exhibited an impressive adsorption capacity for the herbicide 2,4-dichlorophenoxyacetic acid (2,4-D) at 96 ± 6 mg g^–1^ under pH 6 and 25 °C according to the Langmuir–Freundlich model. Notably, N-AC preserves its high adsorption capacity toward 2,4-D from simulated groundwater and runoff from tomato greenhouse, while performance in real samples from Fyris river in Uppsala, Sweden, causes a decrease of only 4–5%. Owing to the one-step process, high yield, annual abundance of kraft lignin, and use of environmentally friendly activating agents, N-AC has substantial potential for large-scale industrial applications.

## Introduction

Carbon materials (CMs) constitute a promising tool in materials science due to their exceptional properties.^1^ In general, they include fullerenes, carbon nanotubes, graphene, carbon fibers^2^ and activated carbon (AC), which are used widely across industries and research laboratories. These materials are essential as catalysts and battery and capacitor components,^3^ as well as in biological, medical, and environmental applications.^4^ The latter area is intensively growing as CMs have been shown to be excellent sorbents for pollutants or contaminants in liquids and gases. ACs dominate the field due to their economic synthesis methods and high efficiency for desired purposes^5^ and have been used to capture CO_2_,^6^ heavy metal ions,^7^ organic contaminants,^7,8^ and various others.

Producing ACs using readily available, low-cost resources like biowaste presents an eco-friendly alternative to the traditional fossil fuel-based production chain.^9^ AC characteristics can be tuned by varying the synthesis conditions, by the utilization of additives and, above all, by the choice of raw materials. Plant-based biomass and byproducts from crops and wood industries (and various others) have attracted attention as potential sources for AC production.^9^ Analyzing the chemical composition of these raw materials, referred to as lignocellulosic biomass, reveals three primary components: cellulose, hemicellulose, and lignin.^10^ Cellulose and hemicellulose generally yield lower carbon content during carbonization, resulting in lower yields. In contrast, the inherently aromatic nature of lignin provides a higher carbon content, leading to increased yields during the carbonization process.^11^ Furthermore, lignin stands as the second most prevalent macromolecule in nature and is readily available as a byproduct of the pulping industry, rendering it an exceptional precursor for AC synthesis.^12^ Various forms of technical lignin exist, including organosolv lignin, lignosulfonates, soda lignin, hydrolyzed lignin, and the most prevalent kraft lignin. Kraft lignin serves as the primary byproduct in the pulping industry. Nevertheless, due to its limited solubility in aqueous solutions (only in alkaline environments) and low reactivity, the majority of it is incinerated for energy production. Although efforts have been undertaken to harness the potential of kraft lignin, it is noteworthy that less than 2% of the approximately 9 million tonnes^13^ produced annually is utilized. The utilization of kraft lignin in the production of AC offers a promising avenue, particularly when prepared in conjunction with chemical activation agents.^14−17^ In this process, lignin is combined with alkali metal hydroxides, salts, or mineral acids and subsequently subjected to carbonization in an inert atmosphere at elevated temperatures. The functionality of AC is significantly improved with the incorporation of heteroatoms, mainly N^18,19^ and S,^18^ in the carbon framework. The simplest method for AC doping involves saturating the modifier into the lignin precursor prior to thermal treatment. Commonly used modifiers include urea,^20,21^ adenine,^19^ NH_3_,^22^ melamine,^23^ chitosan,^24^ and thiourea.^18^

It is a widely established fact that N-doped adsorbents demonstrate superior CO_2_ adsorption capacities when compared to their nondoped carbon counterparts. This phenomenon arises from the synergistic interplay between chemical and physical adsorption processes.^25^ N-containing adsorption centers attract CO_2_ through acid–base interactions, while the tunable microporosity of N-AC captures CO_2_ via physical mechanisms or weak bonding with the solid adsorbent surface.^14^ N-based ACs from lignin have been synthesized using KOH as an activating agent, resulting in intercalated potassium cations in the carbon framework and enhanced adsorption capacities.^26^ However, hydroxide activation results in low carbon yield and has a detrimental impact on the environment.^15^ Activation with phosphoric acid results in the formation of AC characterized by a low microporosity fraction.^15^ This leads to decreased CO_2_ adsorption and selectivity over N_2_ in comparison to other ultramicropore composites.^27^

AC is also an effective sorbent for removal of organic pollutants (herbicides, pesticides, pharmaceuticals, etc.). AC has demonstrated high adsorption capacities toward 2,4-dichlorophenoxyacetic acid (2,4-D),^28−30^ 4-chloro-2-methylphenoxyacetic acid,^29,30^*R*-2-(4-chloro-2-methylphenoxy)propionic acid,^29^ 4-amino-3,5-dichloro-6-fluoro-2-pyridyloxyacetic acid,^29^ 3,5,6-trichloro-2-pyridyloxyacetic acid,^29^ tetracycline,^16^ and many organic compounds. Many of these pollutants are of acidic nature and thus take an anionic form in aqueous environments. It is well known that AC has a pH_zpc_ within the range of pH 4–7.^31,32^ Hence, ACs typically show low adsorption capacities in solutions with a slightly acidic, neutral, or basic medium. Indeed, some ACs prepared from biomass^33^ show high adsorption in very acidic conditions (pH ≤ 3), where the carbon surface has a positive charge and the organic species largely exist in neutral form with no electrostatic repulsion. However, AC adsorbents show a dramatic decrease in adsorption capacity at pH 6–8, a typical pH range for wastewater applications. In comparison, N-based AC sorbents exhibit greater pH resilience. Recent demonstrations have highlighted the effectiveness of N-doped CMs, including CNT^24^ and biochar,^34^ in efficiently removing phenol derivatives such as *p*-nitrophenol (with a capacity of 592.8 mg g^–1^) and acetaminophen (with a capacity of 120.7 mg g^–1^) from aqueous solutions within the pH range of 6–7. Despite the potential of biobased precursors, there has been limited exploration of bioderived N-AC materials for the adsorption of herbicide 2,4-D. Only, Scheufele’s group^35^ has recently reported the development of N-AC derived from fish scales, which exhibited an adsorption capacity of 11.1 mg g^–1^ at pH 6.5 at 30 °C. Notably, this capacity surpassed that of commercial granular AC, which measured at 7.43 mg g^–1^ under similar conditions.

In this study, we employed a metal-free, one-step green chemistry approach to synthesize nitrogen-doped carbons (N-AC) using softwood kraft lignin as the precursor. This synthesis involved the use of nitric acid as an activator and urea as a nitrogen dopant. Urea played a crucial role in introducing nitrogen atoms into the carbon, while the treatment of lignin with nitric acid resulted in the formation of ultramicropores that are highly conductive for CO_2_ adsorption. Our findings suggest that this material holds great potential for applications in carbon dioxide capture as well as the adsorption of 2,4-D from simulated groundwater (SGW), agricultural media, as well as from real surface water.

## Materials and Methods

### Chemicals and Reagents

Kraft lignin (softwood, 100) was supplied by UPM BioPiva, Finland. Urea, copper sulfate, acids (acetic, hydrochloric, nitric, and 2,4-dichlorophenoxyacetic (2,4-D)), sodium hydroxide, sodium carbonate, sodium bicarbonate, and sodium chloride (all ACS Reagent grade) were purchased from Sigma-Aldrich and were used without additional purification.

### Carbonization Process

First, 1 g of lignin was mixed with 200 mg of urea, followed by keeping the mixture at room temperature for 4 h. Next, 2.5 mL of nitric acid (40%) was added to the lignin–urea mixture and carefully mixed with a glass rod. The system was left for 30 min at room temperature, followed by the addition of 100 mg of urea. The obtained mixture was then transferred to a tube furnace and heated under a nitrogen atmosphere to 800 °C with a heating rate of 6 °C min^–1^ and kept at this temperature for 2 h, after which the system was cooled to room temperature. Finally, the obtained product (N-AC) was washed with distilled water and dried in an oven at 60 °C. The described conditions were optimized and discussed below.

### Characterization

#### Initial Kraft Lignin Characterization

The procedures for molecular weight analysis, ^31^P NMR analysis, and thermal gravimetric analysis of initial kraft lignin are described in the Supporting Information.

#### Elemental Analysis

N elemental analysis of samples was performed at Delta Q IRMS with Flash EA for CN percentages.

#### Boehm Titration

The method^36^ was used to determine the basic and acidic surface sites of N-AC. Approximately 0.1 g of N-AC was suspended in 20 mL of a 0.02 mol L^–1^ basic reactant (NaOH, Na_2_CO_3_, or NaHCO_3_) or acidic (HCl) solution. The systems were shaken in an Orbital Shaker INC/REFRIG 5000IR at 130 rpm at 20 °C for 24 h. The solid and liquid phases were separated via centrifugation at 3800 rpm for 10 min. The liquid phase was used in back-titration to determine functional group contents. The filtrate was titrated with the standardized HCl (0.02 mol L^–1^) or NaOH (0.02 mol L^–1^) solutions to calculate the number of acidic and basic sites, correspondingly. According to Boehm, NaHCO_3_ neutralizes only carboxylic groups; Na_2_CO_3_ neutralizes carboxyl and lactone (so lactone content is extracted from the acidity valued by carbonate and bicarbonate); NaOH neutralizes phenolic, carboxyl, and lactone groups (so phenolic content is a difference between the acidity registered with NaOH and Na_2_CO_3_); and HCl gives the total basic content.

#### pH of the Point of Zero Charges

The pH_pzc_ of N-AC was evaluated by applying the pH drift method. The desired initial pH (pH_i_) of the supporting electrolyte solution (0.01 mol L^–1^ NaCl) was adjusted by adding 0.01 M HCl or 0.01 M NaOH. 0.01 g of lignin samples was added to 10 mL of solution and shaken for 48 h. Then, the solid was filtered off, and the final pH (pH_e_) of the solution was measured.

#### FTIR Spectroscopy

Spectra were recorded with a Bruker Tensor 27 ATR-FTIR Spectrometer (Bruker) on dried pellets prepared by pressing the mixture of N-AC (1 mg) and 300 mg of KBr under 8 tons.

#### X-ray Photoelectron Spectroscopy

X-ray photoelectron spectroscopy (XPS) spectra of N-AC were registered on a PHI Quantera II Scanning XPS Microprobe (Physical Electronics) equipped with an X-ray source of monochromatized Al Kα (passing energy 224 and 55 eV for survey and high-resolution spectra, respectively). As the obtained carbon is a conductive material, no neutralization was used. The collected data were examined using MultiPak software (Physical Electronics). The calibration of the binding energy scale was performed by taking as reference the Au 4f peak at 84.0 eV. Gauss–Lorentz peak profiles (90% of Gauss) were used for spectral deconvolution.

#### Powder X-ray Diffraction of N-AC

The spectra were recorded with a D8 ADVANCE Powder Bruker diffractometer using Cu anode (0.15406 nm), Bragg–Brentano geometry in the 2θ range from 10 to 70°, and a step size of 0.017°.

#### Raman Spectroscopy

The analysis was performed on a Horiba LabRAM HR Evolution spectrometer. The spectrum was recorded with a laser of 532 nm and an acquisition time of 2 s.

#### Scanning Electron Microscopy

Images were acquired using a Hitachi SU8230 UHR Cold Field Emission microscope, with an electron accelerating beam of 10 kV. Image analysis was done with Fiji/ImageJ software.

#### Specific Surface Area and Porosity

Nitrogen adsorption–desorption isotherm at −196 °C and carbon dioxide adsorption isotherm at 0 °C were recorded in an ASAP 2020 instrument (Micrometrics). Before each measurement, the sample was degassed at 200 °C for 12 h under a dynamic vacuum. The data were analyzed using the Micromeritics MicroActive software. BET method was used to calculate SSA_BET_ from the N_2_ isotherm. The total pore volume accessible by N_2_ adsorption, *V*_total_, was determined at 0.98*p*/*p*^0^, and micropore volume, *V*_micro_, was estimated from nitrogen isotherm using the *t*-plot method. The micropore size distributions (PSD_micro,N_2__) and (PSD_micro,CO_2__) were calculated from the adsorption branch of both nitrogen and carbon dioxide isotherms by the nonlocal density functional theory (NLDFT) method using the HS-2D-NLDFT heterogeneous surface model.^37^

### Adsorption Measurements

The synthesized N-AC was evaluated as a potential adsorbent for carbon dioxide capture and removal of herbicides from water.

#### Adsorption of Gases

To estimate the applicability of lignin-derived nitrogen-doped carbon for the capture of CO_2_, the material selectivity toward the gases (CO_2_ and N_2_) was calculated using an ideal adsorption solution theory (IAST)^38^ (see more details in the Supporting Information). For this purpose, the individual N_2_ and CO_2_ gas adsorption isotherms were registered at 20 and 25 °C in an ASAP 2020 instrument. The isotherms were fitted with a single-site Langmuir (CO_2_) and Henry (N_2_) models (see the Supporting Information), and the selectivity (*S*) was estimated for a model gaseous mixture containing 15% of CO_2_ and 85% of N_2_ using the equation^39^
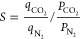
1where *q*_NO_2__ and *q*_N_2__ are amounts of adsorbed gases CO_2_ and N_2_, correspondingly, and *P*_CO_2__ and *P*_N_2__ are the respective pressures.

#### Adsorption of Herbicides

2,4-D herbicide was chosen as a model for the evaluation of N-AC applicability to remove herbicide from water resources. The batch technique was used to investigate the adsorption behavior of N-AC. A precisely weighted adsorbent sample (∼20 mg) was suspended in a solution of 2,4-D (adsorbent dosage 0.8 g L^–1^) at a known initial analyte concentration. The system was shaken in an Orbital Shaker INC/REFRIG 5000IR at 130 rpm and at 25 °C for 24 h (shaking time was only varying in the kinetics study). Then, the solid and liquid phases were separated via centrifugation at 3900 rpm for 5 min, and the residual concentrations of 2,4-D were determined utilizing spectrophotometry in a UV-3100PC spectrophotometer at 283 nm. The removal efficiency (*R*, %) and specific concentration of the adsorbed 2,4-D (*q*, mg g^–1^) were calculated according to
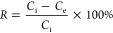
2
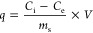
3where *C*_i_ and *C*_e_ are the initial and equilibrium 2,4-D concentrations, respectively, *m*_s_ (g) is the weight of the sorbent, and *V* (L) is the volume of the initial solution. All experiments were repeated at least three times.

The effect of pH was investigated in the presence of 80 mg L^–1^ 2,4-D and in the pH range of 1–10, adjusted by the addition of 0.1 mol L^–1^ HCl or NaOH. The adsorption kinetics was studied in solutions (at pH 6) containing 30 and 100 mg L^–1^ 2,4-D. The equilibrium concentration of 2,4-D was measured within 10 min to 24 h, and the corresponding adsorption (*q*_t_) was calculated according to eq 3. The adsorption capacity of N-AC was evaluated based on the isotherm study. The experiments were carried out at pH 6 with varying concentrations of 2,4-D up to 120 mg L^–1^. The adsorption parameters were calculated according to eqs 2 and 3.

The potential of the practical application of N-AC was evaluated in simulated and real water samples: (1) SGW at pH 6.5,^40^ (2) simulated agricultural wastewater (SAW) from a tomato greenhouse at pH 6.2 (see the Supporting Information, the information about wastewater composition was supplied by the Centre of Expertise Water Technology, Leeuwarden, The Netherlands), and (3) actual natural river water (ARW) at pH 7.3 (Fyris river, Uppsala, Sweden). The experiments were performed at two concentrations of 2,4-D, 40 and 150 mg L^–1^. The precisely weighted mass of the herbicide was dissolved in the target water samples, and the required dilution was performed by the same water sample. The residual concentration of the herbicide was determined using spectrophotometry and the calibration plots made for each water sample media. River water composition is more complex due to the presence of macroelements, microelements, dissolved gases, organic matter, and admixtures. Therefore, to simplify the calculation procedures, the analysis of ARW was performed with the assumption that the natural water matrix components have a higher affinity to N-AC than 2,4-D herbicide. For this, prior to the experiment with ARW with added 2,4-D, the adsorption experiments were performed without the addition of 2,4-D to the ARW sample. Then, the absorbance used for calculations was the difference between the absorption of ARW with added 2,4-D and ARW without added 2,4-D.

### Theoretical Models for Adsorption Fitting

#### Adsorption Kinetics and Equilibrium Models for Fitting 2,4-D Data

The kinetic data were fitted by using four adsorption kinetic models [pseudo-first-order (PFO), pseudo-second-order (PSO), mixed 1,2-order equation (MOE), and intraparticle diffusion]. The equations of the applied models’ are as follows:

The PFO
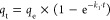
4

The PSO
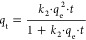
5

The mixed 1,2-order
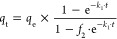
6

Weber and Morris equation
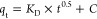
7where *q*_t_ and *q*_e_ (mg g^–1^) are the specific concentrations of 2,4-D adsorbed at time *t* (min) and equilibrium, *k*_1_ (min^–1^) and *k*_2_ (g mg^–1^ min^–1^) are PFO and PSO rate constants, respectively, *K*_D_ is the intraparticle diffusion rate (mg g^–1^ min^–0.5^), and *C* is a constant (mg g^–1^).

The experimental equilibrium data were fit by applying Langmuir (8), Freundlich (9), and Langmuir–Freundlich (10) models
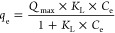
8
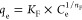
9
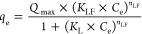
10where *Q*_max_ (mg g^–1^) is the maximum adsorption capacity of N-AC; *K*_L_ (L mg^–1^), *K*_F_ (*L*^1/*n*_F_^ mg^1–1/*n*_F_^ g^–1^), and *K*_LF_ (L mg^–1^) are Langmuir, Freundlich, and Langmuir–Freundlich equilibrium constants; and *n*_F_ and *n*_LF_ are the dimensionless exponents of Freundlich and Langmuir–Freundlich models related to the heterogeneity of the adsorption process. Nonlinear fitting of equilibrium data was performed using the Microcal Origin (2019) software.

To verify the statistical adequacy of the models, the adjusted determination coefficient (*R*_adj_^2^), standard deviation of residues (SD), and Bayesian information criterion (BIC)^34,41^ were calculated according to equations
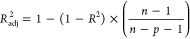
11
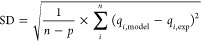
12
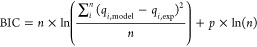
13where *n* is the total number of experimental points, *p* is the number of fitting parameters, and *q*_*i*,model_ and *q*_*i*,exp_ are calculated and measured properties, respectively (*q*_t_ for kinetic and *q*_e_ for equilibrium models). The *R*_adj_^2^ values closer to 1.0 and the lowest SD are attributes of the best-fitted model. Parameter BIC is used to elucidate whether the difference between two models with very close *R*_adj_^2^ and SD is significant or whether both models provide fitting within the permissible deviations.^42^ The difference in BIC values lower than 2 means no significant difference between the models. When it lies within 2–6, then the model with lower BIC shows a positive perspective for fitting. If the BIC values of the two models differ within 6–10, there is a strong possibility the model with a lower BIC value would be the best one to approximate data. Finally, if variations of BIC values are higher than 10, we can predict that with accuracy the model with the lower BIC value provides better fitting.

## Results and Discussion

### Material Characterization

#### Preparation of N-AC through Pyrolysis Carbonization

Pyrolysis carbonization of lignin includes several parallel or consecutive steps which are the superposition of primary mechanisms (char formation, depolymerization, and fragmentation) and secondary mechanisms (cracking and/or recombination).^10,43,44^ AC properties are highly dependent on pyrolysis conditions such as activating agent, temperature and holding time, heating rate, and inert atmosphere flow rates. Therefore, to obtain reproducible N-AC material with desired characteristics, the pyrolysis conditions were optimized or selected based on published works.

Bouchelta et al.^45^ studied how pyrolysis conditions effect the structure of porous AC obtained from date pits. The highest total volume and surface area were obtained for the following parameters: 700–800 °C carbonization temperature, 100–150 cm^3^ min^–1^ of nitrogen flow rate, and 2–4 h of holding temperature. Since lignin material has fewer volatile matters than date pits (where both cellulose and hemicellulose are major components), we used a nitrogen flow rate of 100 cm^3^ min^–1^ to provide the extraction of volatile compounds at the appropriate level and prevent the unnecessary temperature reduction of char particles. Considering that Bouchelta et al. found only a minor change in the total pore volume when the holding time was increased from 2 to 4 h as well as the energetic cost of heating, 2 h was selected as the optimal time for lignin carbonization. Fujita et al.^46^ used urea as a coactivating and modifying agent to obtain N-AC from Shochu waste. According to their results, 5 °C min^–1^ heating rate provided the highest N-content in N-AC, the highest surface area, and the greatest total pore volume. Considering that Shochu waste is cellulose based, we applied 6 °C min^–1^ heating rate for the lignin carbonization to increase the rate of changes in the lignin. The above parameters and the amounts of activating and modifying agents as reported were used as a basis to investigate the influence of carbonization temperature on the porosity of N-AC.

Considering the TGA results of initial kraft lignin (Supporting Information), where the plateau became visible starting from 700 °C (38.4% of the residual solid content), the N-AC samples were prepared at three temperatures: 700 °C, 800 °C (36.0% solid content), and 900 °C (35.4% solid content), and the samples were assigned as N-AC(700), N-AC(800), and N-AC(900), correspondingly. The comparison analysis of the N-AC ability to adsorb liquid nitrogen was estimated through the amount of nitrogen adsorbed at three values of *p*/*p*^0^: 0.05, 0.16, and 0.98. The obtained results are presented in Table S3. The amount of nitrogen adsorbed at 0.05*p*/*p*^0^ is related to the volume of micropores.^41^ As could be seen, samples obtained at higher temperatures showed higher adsorption capacity (12.0 and 13.0 mmol g^–1^ for N-AC(800) and N-AC(900), respectively) than N-AC prepared at 700 °C (5.8 mmol g^–1^), indicating that higher temperatures yield materials with a larger number of micropores. Further analysis showed that these samples also adsorbed larger volumes of nitrogen at higher *p*/*p*^0^, 0.16 and 0.98 (12.5 and 12.8 mmol g^–1^ for N-AC(800) and 14.1 and 16.6 mmol g^–1^ for N-AC(900)), while N-AC(700) had a minor enhancement in the capacity (5.9 and 6.2 correspondingly). The results indicated that N-AC(700) was a microporous material, while N-AC(800) and N-AC(900) had potentially micro- and mesoporous structures. Considering the target applications, N-AC(800) and N-AC(900) were more attractive. However, the sample obtained at 900 °C had a very low yield (appr. 2%), while N-AC(800) showed 30%. In addition, the benefits of utilizing 800 °C as the most optimal temperature for kraft lignin carbonization were demonstrated through adsorption test of CO_2_ and N_2_ at room and normal conditions (see Supporting Information). Considering all statements presented above, 800 °C was chosen as the optimal temperature for the carbonization process.

The nature and quantity of activating and modifying agents are other important factors in the formation of N-AC. H_3_PO_4_ is a widely used nonmetal activating agent for AC preparation via a chemical activation technique,^17^ while nitric acid is usually applied for postcarbonization treatment to increase surface functionality. Nonetheless, several works have attempted to use nitric acid as the activating agent. Ngamthanacom et al.^47^ obtained AC from water-soluble lignin using 5% solution of HNO_3_. The preparation process was performed through three steps (dissolving of lignin in solution of HNO_3_, drying in oven at 100 °C, followed by the carbonization at 800 °C for 2 h). The obtained AC had a surface area of 744 m^2^ g^–1^, but the authors did not provide the information about the structure of pores. Pakade et al.^48^ investigated the influence of inorganic acid type and solution concentration on the pore volume and surface area during the activation of Macadamia AC. In the case of nitric acid, the following concentrations were studied 20, 40, and 55%. The obtained textural parameters were found to be 0.369, 0.373, and 0.388 cm^3^ g^–1^ (pore volume) and 565, 582, and 583 m^2^ g^–1^ (surface area) for the stated above concentration. Considering the minor increase in pore volume and the nearly insignificant change in surface area when increasing from 40 to 55% nitric acid, a 40% solution of nitric acid was used for the impregnation of kraft lignin as described in Materials and Methods.

Pietrzak^49^ and Zhan^50^ successfully applied urea for carbon activation and functionalization with nitrogen groups. The optimal ratio between the activating agent and carbon was 1:1. The obtained N-containing carbons were micromesopore materials with a total nitrogen content of 4–5%. Since the thermal treatment of kraft lignin at 800 °C led to the formation of a solid carbon matrix, which was 30% of the total mass of the initial kraft lignin sample (TGA results in the Supporting Information), and considering the results of Pietrzak and Zhan, we chose ratio 1:0.3 as the most optimal ration for lignin carbonization.

In summary, based on the literature analysis and simple experiments, the carbonization conditions presented in Materials and Methods were selected as the most optimal, and the obtained product was assigned as N-AC in the following experiments.

For comparison, carbons without any pretreatment were prepared under the same conditions as N-AC. Liquid nitrogen adsorption showed the material to be nonporous with adsorption values of 0.005 mmol g^–1^ (0.05*p*/*p*^0^), 0.011 mmol g^–1^ (0.16*p*/*p*^0^), and 0.014 mmol g^–1^ (0.98*p*/*p*^0^). The results indicate that impregnation of lignin with nitric acid and urea before thermal treatment was effective and led to the carbonization and activation in a one-step process. The obtained N-AC material underwent further detailed characterization before investigating liquid and gas adsorption behavior for targeted applications.

The presented method exhibits significant advantages in terms of both yield and environmental impact. Importantly, the proposed method represents a one-step route, distinguishing it from other established approaches for the synthesis of N-containing carbons. Unlike conventional methods that usually require at least two high-temperature steps—either carbonization followed by N-doping or vice versa—the simplicity and efficiency of this method underscore its potential for practical applications. The use of KOH as an activating agent was shown to result in a carbon output ranging from 3 to 40%, depending on variables such as the type of lignin, the quantity of catalyst, and the carbonization temperature.^14,51,52^ Due to the multistep carbonization process for N-containing carbons outlined in Table S4, authors often omitted information about the yield. Consequently, a cost analysis for the fabrication of 1 and 5 kg adsorbent was conducted, assuming the 20% (as an average) carbon yield for materials lacking yield data and 30% for N-AC (as discussed earlier). The results are presented in Table S5. Notably, the preparation of N-AC emerges as a significantly less time-consuming and energy-intensive process that requires fewer additional reagents, both as an activating agent and a N-dopant. The estimated reagent expenses for the preparation of 1 and 5 kg of N-AC are 282.6 and 682.1 USD, respectively. In contrast, other N-containing carbons with similar adsorption behavior but prepared using adenine, melamine, or thiourea incur costs ranging from approximately 450 to 74,000 USD (for more details, refer to Table S5) and necessitate 3–4 times more time for carbonization, inevitably escalating the overall production costs.

#### Elemental Analysis

The elemental analysis was conducted to confirm the efficacy of our methodology in incorporating N atoms in the structure of N-AC. A comparison of N elemental content in samples N-AC, initial kraft lignin, and carbon without any pretreatment revealed that no nitrogen atoms were detected in kraft lignin and carbon. In contrast, N-AC exhibited a N content of 3.5%. Importantly, our proposed method enables the incorporation of a larger or comparable number of nitrogen atoms using a smaller amount of nitrogen agent in a one-step procedure. In contrast, similar materials obtained through the chemical activation route (Table S4) typically require two- or three-step operations.

#### Boehm Titration

In general, the obtained N-AC is a material with a relatively low number of functional groups on the surface. The detected specific concentrations of the carboxylic and N-heterocyclic/amine groups did not exceed the experimental uncertainties (0.03 ± 0.03 mmol g^–1^). This might be related to the absence of carboxylic and N-based groups with p*K*_a_ < 6.4. The second acidic group (lactonic, low p*K*_a_ phenolic, or N-heterocyclic/amines with p*K*_a_ 6.4–10.3) was detected at the level of 0.05 ± 0.02 mmol g^–1^. The last group (phenolic, N-heterocyclic/amines with p*K*_a_ 10.3–13) was determined at the level of 0.22 ± 0.02 mmol g^–1^. The total acidic and basic contents estimated with titration by NaOH and HCl, respectively, were found to be 0.21 ± 0.03 mmol g^–1^. However, it should be noted that Boehm’s titration does not allow the quantification of aliphatic hydroxyl groups.

#### pH of the Point of Zero Charges

**Figure 1:**
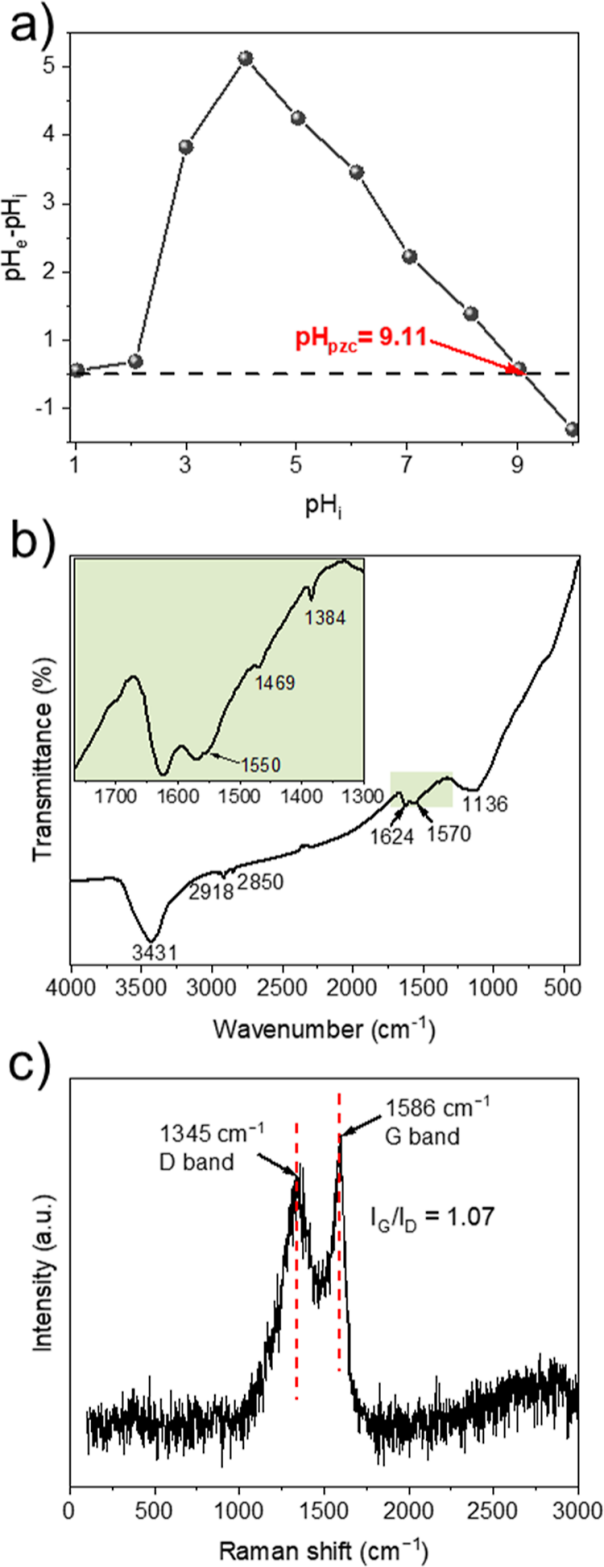
Surface and functional characterization of N-AC: (a) pH_pzc_; (b) FTIR spectrum; and (c) Raman spectrum.

The obtained pH drift of a water suspension of N-AC vs pH_i_ is presented in Figure 1a. The estimated value of pH_pzc_ was found to be 9.11. This high value indicates that N-AC is a positively charged adsorbent in a wide pH range. Considering that lignin-based adsorbents^53^ (which are rich in aliphatic and phenolic OH groups) and ACs^31,32^ have pH_pzc_ values typically within the range of 4–7, the positive charge in this work is expected due to the incorporation of nitrogen atoms in the structure of N-AC. From the results of pH_pzc_ and Boehm titration, we can assume that N-AC does not have carboxylic groups (and N-heterocyclic or amines with p*K*_a_ < 6.4) and that lactonic, phenolic, and potential aliphatic OH are only present as a minority compared to the nitrogen-containing species. As a result, the N-AC material can be classified as weakly acidic and strongly basic N-containing anion exchangers. This type of adsorbent has great potential for the adsorption of anionic organic species.^54^ To better identify specific functional groups of N-AC, spectroscopy (FTIR, Raman, and XPS) was utilized.

#### FTIR Spectroscopy

Figure 1b shows the FTIR spectrum of N-AC. The strong and broad band at 3431 cm^–1^ was identified as O–H stretching, possibly due to the presence of hydroxyl (aliphatic and phenolic) groups and adsorbed water molecules. The doublet at 2918 and 2850 cm^–1^ was assigned to asymmetric and symmetric stretching of CH_2_ groups. There was no characteristic band for the carbonyl group (which has strong intensity at around 1750 cm^–1^), indicating that N-AC is a CM without COOH, in agreement with Boehm’s titration results discussed above. In addition, the lack of bands in this region further suggests that N-AC does not have lactonic groups, indicating that the value of the second acidic group as determined by Boehm’s titrations should be considered as only phenolic groups with low acidity and N-containing groups. The sharp band at 1624 cm^–1^ and the low intensity peak at 1425 cm^–1^ were assigned to the vibrations of C=C in aromatic rings.^55^ Additionally, adsorbed water might contribute bending vibrations to the peak at 1624 cm^–1^.^41^ The band at 1570 cm^–1^ can be assigned to C–N vibrations in heterocyclic aromatic rings, as well as aromatic skeletal vibrations in C=C. The presence of N–O stretching observed at 1550 cm^–1^ indicates that a oxidized form of nitrogen atom is potentially present in N-AC. The peak at 1380 cm^–1^ (assigned to CH_3_ bending^41^) and doublet CH_2_ bending revealed the presence of aliphatic species fraction (defects). The band at 1380 cm^–1^ was also attributed to O–H bending in phenolic fragments.^55^ A strong and broad band appeared at 1184 cm^–1^ was attributed to C–O stretching vibrations confirming the presence of primary alcohols and phenolic groups.^55^

#### XRD Analysis

The obtained XRD pattern (Figure S3) indicated that N-AC is an amorphous CM that contains graphite-like domains. The broad diffraction peak at 43.6° is assigned to the carbon facets (101); however, the peak of low intensity at 26.6 is referred to graphite (002) planes. The appearance of another small diffraction peak at 25.6 could be related to the incorporation of N atoms in the graphite structure. To confirm this assumption, Raman spectroscopy and XPS were applied.

#### Raman Spectroscopy

The Raman spectrum of N-AC is presented in Figure 1c. Two clear bands were observed at 1345 cm^–1^ (D-band) and at 1586 cm^–1^ (G-band), which were attributed to planar sp^2^ carbon bonds and tetrahedral carbons, respectively.^41^ The degree of graphitization can be estimated by the relative intensities of these bands’ *I*_G_/*I*_D_ ratio, which was found to be of 1.073, reflecting a good degree of graphitization and a more ordered structure. The presence of D-band from Raman spectroscopy was in good agreement with FTIR results, where asymmetric and symmetric stretching of CH_2_ groups were detected.

#### X-ray Photoelectron Spectroscopy

**Figure 2:**
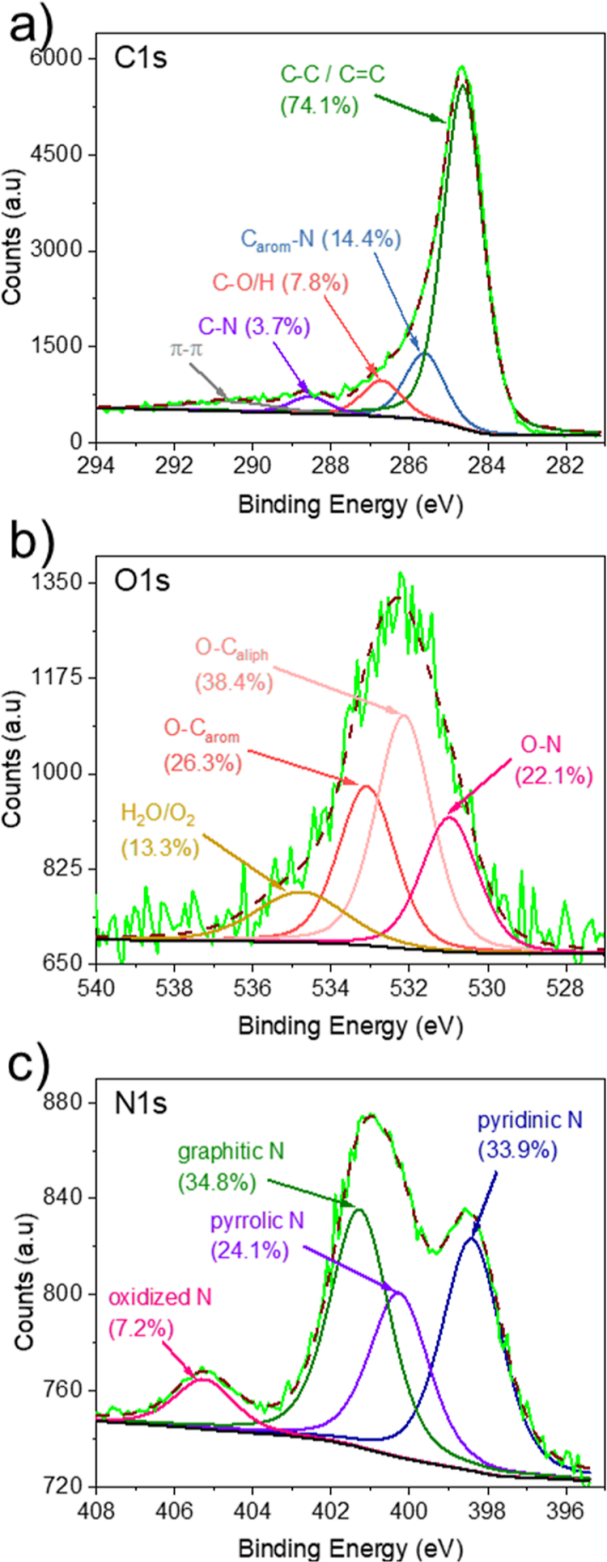
Surface and functional characterization of N-AC: (a) C 1s, (b) O 1s, and (c) N 1s XPS spectra.

To investigate the surface chemistry of N-AC and to evaluate the elemental composition, as well as chemical and electronic states of the atoms, XPS analysis was performed. The survey analysis showed relative elemental composition contents to be 86–92% of C, 5–10% of O, and 4–9% of N by atoms. Figure 2a–c presents the deconvoluted C 1s, O 1s, and N 1s spectra.

The C 1s XPS spectrum exhibited six subpeak components with binding energies of around 284.6, 285.6, 286.7, 288.5, and 290.8 eV. Considering the previously obtained data and N 1s XPS fitting (see below), these peaks correspond to the following groups: C=C/C–C (74.1%), C_arom_–N (14.4%), C–O/H (7.8%), C–N (3.7%), and π–π, respectively.^49^ The appearance of π–π peak as well as shifting the main peak for C 1s to lower energy indicates the highly π-polycyclic aromatic structure of N-AC. It should be noted that a significant portion of C atoms was present in aromatic rings with nitrogen atoms. The deconvolution of the N 1s spectrum presented four subpeak components: pyridinic (at 398.4 eV), pyrrolic (400.2 eV), graphitic (401.2 eV), and oxidized (405.2 eV).^56^ Graphitic N accounts for 35% of all N atoms, while pyridinic and pyrrolic N and oxidized N make up 34, 24, and 7.2%, respectively. This relatively high content of basic nitrogen atom in N-AC explains the relatively high pH_pzc_. As could be seen from the C 1s spectrum, there were no peaks for carbonyl and carboxylic groups. To verify this, the O 1s spectrum was deconvolved. Four main subpeak components were observed with binding energies around 530.9, 532.2, 533.0, and 534.7 eV, which were attributed to O–N (22.1%), O–C_aliph_ (38.4%), O–C_arom_ (26.3%), and H_2_O/O_2_ (13.3%),^57,58^ respectively. The first peak confirms the presence of oxidized nitrogen atoms, in agreement with N 1s peaks from XPS and FTIR data. N-AC was observed to be hydrophilic, which is expected to be the result of hydroxyl groups on the surface, as measured by peaks of O–C_aliph_/O–C_arom_, or the result of absorbed water on the surface of N-AC which was confirmed by the presence of respective peak in O 1s and bands in FTIR spectrum.

#### Scanning Electron Microscopy

**Figure 3:**
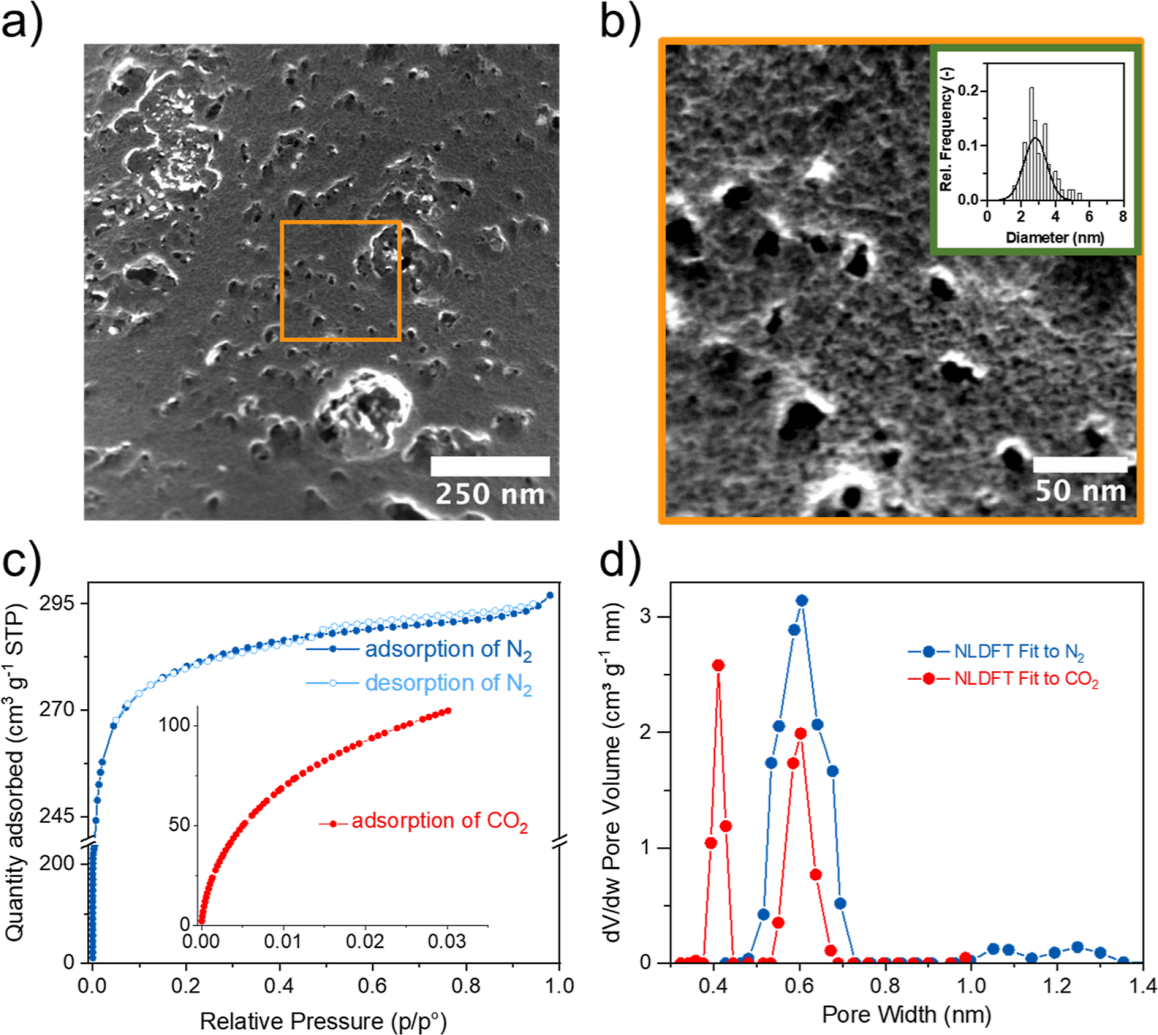
Textural and morphological properties of N-AC: (a,b) SEM micrographs of N-AC. Inserted: pore size distribution onto the surface of N-AC for sub 10 nm pores, *N* = 150; (c) N_2_ adsorption–desorption isotherms at −196 °C and CO_2_ adsorption isotherm at 0 °C; and (d) pore size distribution of N-AC obtained by NLDFT.

SEM images and SEM-based pore sizing are presented in Figure 3b. The surface of N-AC is sparsely covered with pitted cavities of 10–30 nm size range, as well as smaller ones with a mean pore size of 3 nm (*N* = 150, 1.5–6 nm range). We could assume that these cavities turn into micropores deeper inside the bulk matrix. To investigate the presence and distribution of micropores in N-AC, the textural characteristics were investigated via adsorption–desorption of N_2_ and CO_2_.

#### SSA and Porosity

The textural properties of the N-AC material were investigated via nitrogen adsorption–desorption isotherm at −196 °C and carbon dioxide adsorption isotherm at 0 °C. The N_2_ adsorption isotherm shape (Figure 3c) exhibited a steep type I isotherm^59^ with a major uptake at pressures lower than 0.05*p*/*p*^0^. Slight hysteresis observed at above 0.5*p*/*p*^0^ is related to the tensile strength effect.^60^ These features indicate that N-AC is the micropore material having a fraction of large pores with very small neck. The calculated SSA_BET_ value was estimated to be up to 1000 m^2^ g^–1^. The total pore volume was found to be 0.46 ± 0.01 cm^3^ g^–1^, and micropores contributed almost 86% to the total pore volume (0.38 ± 0.03 cm^3^ g^–1^). Jagiello et al.^37,61^ demonstrated that simultaneously evaluating the nitrogen and carbon dioxide adsorption branches leads to a more comprehensive analysis of microporous carbons. Specifically, nitrogen is typically restricted from filling the small pores (width less 0.5 nm), whereas the upper limit for CO_2_ analysis ranges from 0.7 to 1.1 nm.^62^ As a result, measuring the adsorption of each of these gases allows for better interpretation of pore size distribution. The presented pore size distribution obtained by NLDFT method (Figure 3d) shows micropores with a diameter of 0.6 ± 0.05 nm, from the N_2_ isotherm, and pores with a diameter of at 0.41 ± 0.01 and 0.61 ± 0.01 nm from CO_2_ analysis. These results for N-AC are in line with other reported carbons.^37,61^

#### Doping Mechanism

**Figure 4:**
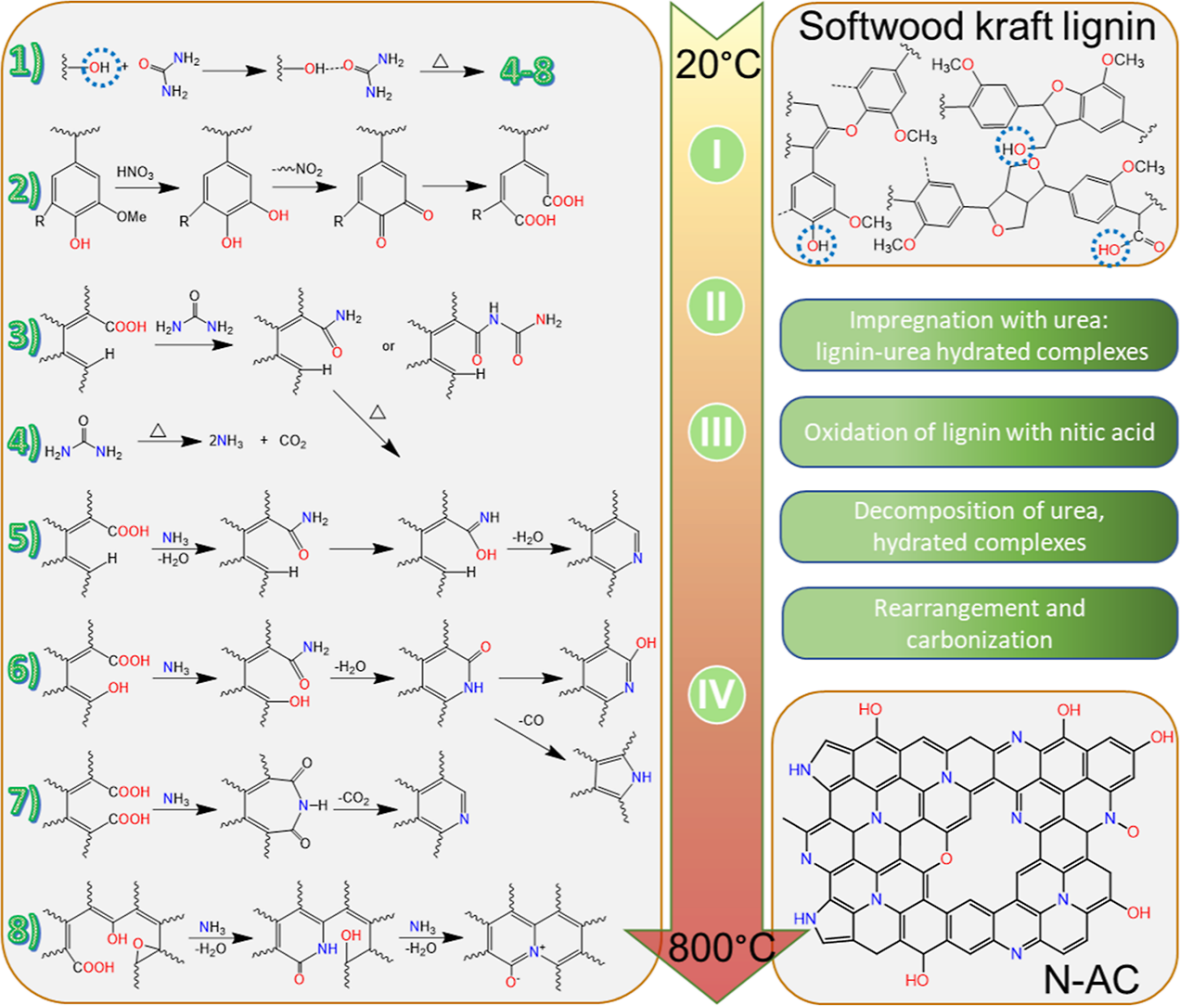
Schematic illustration of lignin transformation in N-doped activated carbon.

The characterization results of N-AC clearly indicate that nitrogen is incorporated into the porous carbon structure. There are possible pathways of lignin pyrolysis;^10,43,44^ here, the nitrogen doping and carbonization of kraft lignin are proposed in Figure 4. Urea has the ability to break hydrogen bonds within the lignin macromolecule (between hydroxyl groups as donors and carbonyl and ether groups as acceptors) and form new hydrogen bonds between carbonyl oxygen in urea and hydroxyl hydrogen on lignin.^63^ Impregnating lignin with wet urea leads to the formation of lignin–urea hydrated complexes through C–O–H atoms (step 1). Subsequent treatment with nitric acid (step 2) could lead to the oxidation of short organic chains and phenol hydroxyls, increasing the number of carboxylic groups.^64^ This process is favored when the temperature increases. It should be noted that nitration of lignin can also take place as oxidized nitrogen was detected in the structure of N-AC. Addition of another portion of urea is used in reactions with newly formed carboxylic groups (step 3). Heating decomposes the urea (step 4) which evolves ammonia or transforms lignin–urea hydrated complex to an amid, followed by intermediates and final N-based fragments (steps 5–8). Additional reactions may occur in the described system as urea decomposition is a several-stage process which can lead to the formation of biuret, triuret, cyanuric acid, and ammelide.^65^ Adding to the complexity the lignin matrix is simultaneously undergoing pyrolysis.^10,43,44^

### CO_2_ Capture

**Figure 5:**
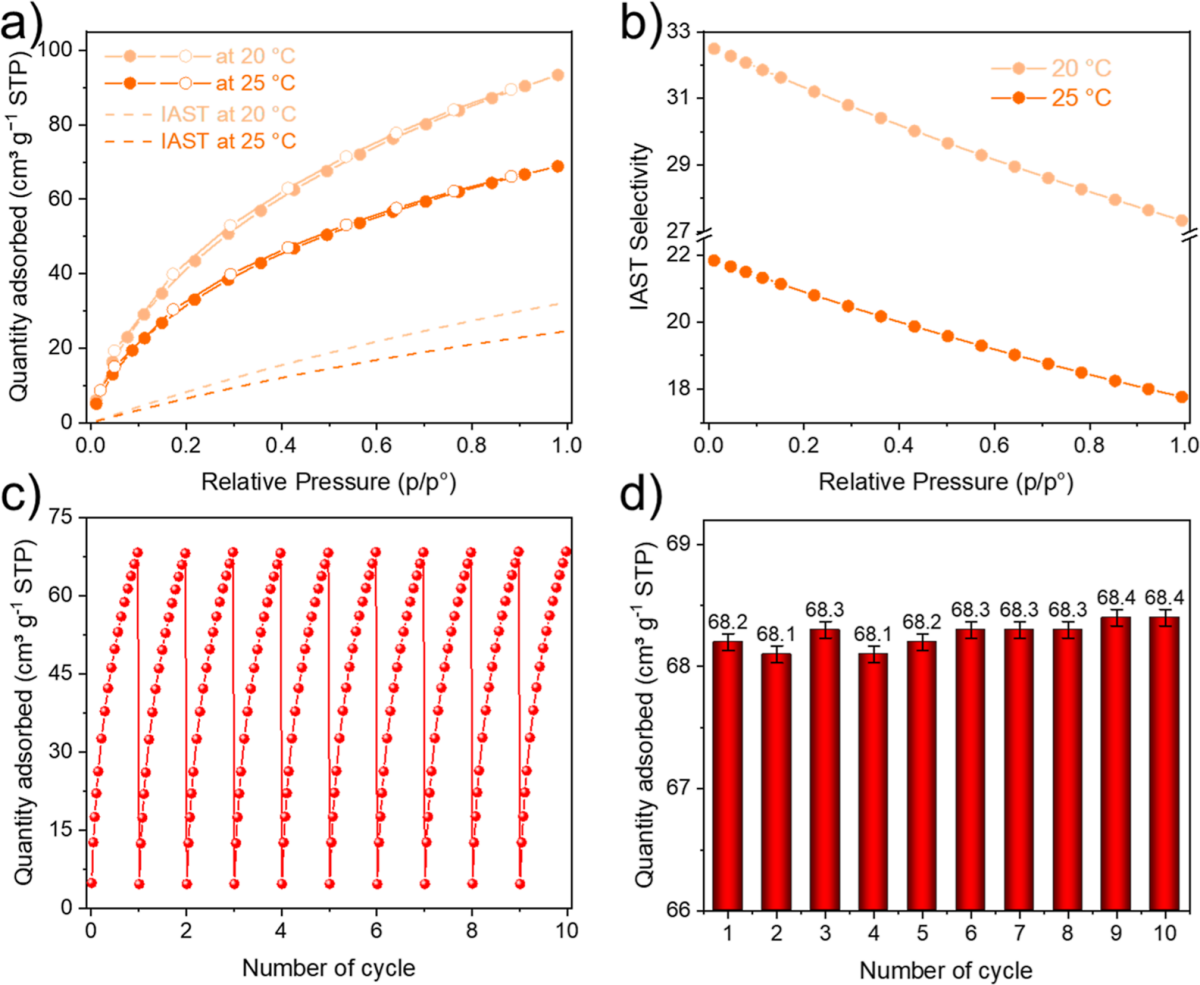
(a) Adsorption (filled symbols) and desorption (hollow symbols) isotherms of CO_2_ at 20 and 25 °C (solid lines) and simulated adsorption IAST isotherms of CO_2_ (dash lines) for gaseous mixture (15% of CO_2_ and 85% of N_2_); (b) CO_2_/N_2_ IAST selectivity of N-AC at 20 °C and at 25 °C for artificial gaseous mixture consisting of CO_2_ (15%) and N_2_ (85%); (c) cyclic study of CO_2_ adsorption on N-AC at 25 °C and at pressure within 0–0.98 bar; and (d) CO_2_ adsorption capacity of N-AC at 25 °C and 1 bar during 10 consequently adsorption–desorption cycles.

Considering the high pyridinic content (approximately 1.15 mmol g^–1^) and microporous nature of N-AC, the prepared material was tested for CO_2_ capture at two temperatures, 20 and 25 °C, under atmospheric pressure (1 bar). The pressure and temperatures were chosen in order to evaluate the efficiency of the material as an air filter and/or CO_2_ sorbent for further valorization. Figures 5b and S4 show single-component CO_2_ and N_2_ adsorption isotherms at both temperatures. It was found that N-AC demonstrated better adsorption ability to CO_2_ than N_2_. The adsorption was enhanced with increased pressure; however, the adsorption capacities decreased when temperature increased from 20 to 25 °C. This behavior is due to the exothermic characteristic of the adsorption process, which is common for CO_2_ adsorbents. The estimated adsorption capacities were 4.2 and 3.1 mmol g^–1^ at 20–25 °C, respectively, comparable with other recently developed CO_2_ adsorbents (see Table S4). However, the high adsorption capacities at 1 bar, demonstrated by many carbons from Table S4, are not representative of a flue gas environment, where CO_2_ content varies from 10 to 15%, which could be represented by 0.1–0.15 bar partial pressure from pure CO_2_ isotherm. In this range CO_2_ uptake by N-AC varied from 1.5 to 1.2 mmol g^–1^ when the temperature increased from 20 to 25 °C, surpassing other adsorbents (Table S4). In addition to the good performance, the manufacturing process of N-AC requires the only one step of high-temperature treatment, while other N-carbons owning slightly higher CO_2_ capture values often require a minimum of two steps.

Since CO_2_ selectivity over N_2_ is a crucial factor for the practical applicability of the adsorbent, we calculated the selectivity of N-AC. First, the *S* values were estimated from single-component isotherms using the eq 1 and were found to be 26.4 and 17.2 at a total pressure of 1 bar and 20–25 °C, respectively. In addition, IAST theory was used to calculate the ideal CO_2_/N_2_ selectivity of N-AC for gaseous mixture (15% CO_2_ and 85% N_2_) and at total pressure range from 0.01 to 1 bar for both temperatures. The parameters used to fit single-component gas uptakes as a function of pressure are presented in Table S6. The simulated IAST isotherms of gases (Figures 5a and S4) indicate CO_2_ capacities of 1.4 and 1.1 mmol g^–1^ at 20 and 25 °C, respectively, which are consistent with the values obtained from the single CO_2_ isotherms. The calculated *S* values (Figure 5b) demonstrate a slightly higher separation performance of N-AC at lower pressure for both temperatures. The values varied from 32.5 (0.1 bar) to 27.3 (1 bar) and from 22.8 (0.1 bar) to 17.8 (1 bar). The selectivity of N-AC was comparable or even higher than that of other N-doped carbons from biomass (see Table S4), potentially related to the synergetic effect of the high nitrogen content and the ultramicroporosity of N-AC.

The cycling performance of the adsorbent is an important requirement in industrial applications. As can be seen in Figure 5a, the desorption branches of the isotherms show that zero loadings of CO_2_ are reached under low pressure, indicating the excellent renewability of N-AC. To improve the kinetics of the desorption process, degassing at higher temperatures could be applied. The recyclability of the N-AC material was checked through 10 consequent adsorption–desorption cycles. The presented isotherms (Figure 5c) and histograms (Figure 5d) show that N-AC keeps CO_2_ capture level without visible decay. A similar behavior was observed for capacities at 0.15 bar (Figure S5).

### Adsorption of 2,4-D Herbicide

#### Influence of pH

**Figure 6:**
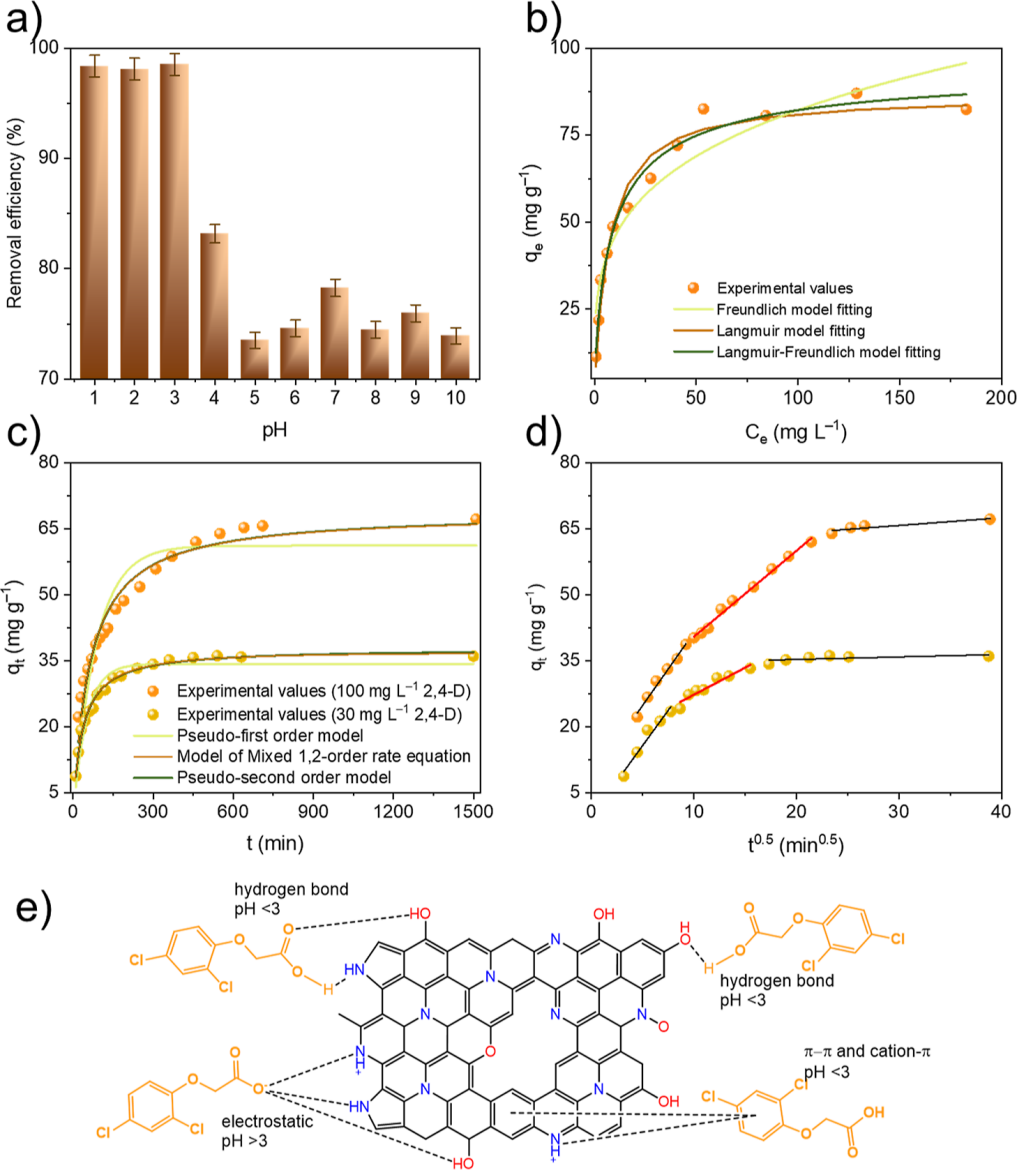
(a) Influence of pH on the adsorption of 2,4-D onto N-AC; (b) adsorption isotherms of 2,4-D (symbols) and nonlinear fitting by the equilibrium models (lines); (c) influence of contact time on 2,4-D adsorption (symbols) and nonlinear fitting by the kinetic models (lines); (d) intraparticle diffusion model fitting of 2,4-D adsorption; and (e) possible adsorption mechanism of 2,4-D onto N-AC. Note: the symbol size on figures (b–d) is equivalent to experimental errors.

N-AC is positively charged in aqueous solutions within a wide pH range (from acidic medium to pH 9). Therefore, the removal efficiency of anionic 2,4-D could depend on its charge in solution. The p*K*_a_ value of 2,4-D is 2.87, indicating that the anion form dominates in solutions with pH higher than 2.87. It was reported that electrostatic interaction, π–π conjugation, or donor–acceptor bonding could be possible mechanisms involved in the adsorption of 2,4-D.^66^ The effect of pH on 2,4-D adsorption onto N-AC is presented in Figure 6a. The removal efficiency reached 98.5% when adsorption occurred from very highly acidic solutions (pH 1–3); however, it dropped to 83% for pH 4. The *R* values were 73.5–78.2% for weakly acidic, neutral, and weakly basic media. Ionic attraction was a driving force within the pH range 4–9 in the adsorption process when both 2,4-D and N-AC have opposing charges. However, the high value of *R* for pH 10 could be a result of the buffering capacity of N-AC adsorbent. According to the pH_pzc_ study, N-AC increased the initial pH of the solution from 10 to 9 when suspended in water for 24 h. Potentially the same phenomenon occurred during the adsorption of 2,4-D from a solution with pH 10. While the value of pH_pzc_ was 9.18, both 2,4-D and N-AC were still oppositely charged and thus maintained electrostatic interactions. The higher adsorption in the pH range 1–3 might be attributed to hydrogen bonding between molecular form 2,4-D and hydroxyl groups of N-AC surface, as well as to π–π or cation_N-AC surface_–π conjugations. Considering that N-AC is rich of aromatic rings, a number which is higher than the number of positively charged centers, adsorption is more effective from highly acidic media due to a greater number of interactions. All possible adsorption pathways are presented in Figure 6e. TGA was applied for the determination of the lignin layer weight concentration in the composite materials and to evaluate the thermal properties of the composites. TG-curves for composites as well as pure silica, KL, and LS are depicted in Figure 2.

#### Equilibrium Studies

Since the pH of groundwater typically ranges from about 6.0 to 8.5, the solutions of 2,4-D at pH 6 were chosen for both adsorption equilibrium and kinetics studies. The obtained adsorption isotherm is presented in Figure 6b. The experimental adsorption capacity was found to be 85 ± 2 mg g^–1^. The obtained curve was fit with Langmuir, Freundlich, and Langmuir–Freundlich isotherm models, and the results of the fitting are presented in Figure 6b and Table 1. The statistical parameters SD and *R*_adj_^2^ demonstrated that Langmuir and Langmuir–Freundlich models better described the experimental data with the Freundlich model being less suitable. This is also confirmed by the BIC parameters, where both Langmuir and Langmuir–Freundlich models had lower values compared to the Freundlich model (differences were 14 and 17, correspondingly). When comparing the Langmuir and Langmuir–Freundlich models, fitting parameters and the difference of BIC parameters (near three unities) indicate that the Langmuir–Freundlich model better represents the data. The sufficient correlation of experimental data to the Langmuir–Freundlich isotherm model could be a result of the complicated adsorption process that occurred on the surface of N-AC since the isotherm model is based on assumptions of the monolayer adsorption model with the notable impact of surface heterogeneity as well. Thus, N-AC could be considered as heterogeneous adsorbent which is composed of energetically different sites distributed throughout the surface.^67^

**Table 1:** Results of Fitting of Equilibrium and Kinetics Adsorption of 2,4-D onto N-AC

Parameter equilibrium models	
Langmuir model	
*Q*_max_, mg g^–^^1^	87 ± 3
*K*_L_, L mg^–^^1^	0.14 ± 0.02
*R*_adj_^2^	0.969
SD, mg g^–^^1^	4.53
BIC	39
Freundlich model	
*K*_F_, L^1/*n*_F_^ mg^1–1/*n*_F_^ g^–1^	25 ± 3
*n*_F_	0.25 ± 0.03
*R*_adj_^2^	0.901
SD, mg g^–^^1^	8.06
BIC	53
Langmuir–Freundlich model	
*Q*_max_, mg g^–^^1^	96 ± 6
*K*_LF_, L mg^–^^1^	0.11 ± 0.03
*n*_LF_	0.76 ± 0.09
*R*_adj_^2^	0.978
SD, mg g^–^^1^	3.79
BIC	36

Parameter kinetics models		
	concentration of 2,4-D, mg L^–^^1^	
	30	100
*q*_e,exp_, mg g^–^^1^	36.0 ± 0.1	67 ± 1
Pseudo-first-order model		
*q*_e,cal_, mg g^–^^1^	34.3 ± 0.3	61 ± 2
*k*_1_, min^–1^	0.020 ± 0.002	0.012 ± 0.001
*R*_adj_^2^	0.935	0.859
SD, mg g^–^^1^	2.1	5.32
BIC	29	67
Pseudo-second-order model		
*q*_e,cal_, mg g^–^^1^	37.8 ± 0.4	69 ± 2
*k*_2_, g mg^–^^1^ min^–^^1^	0.029 ± 0.001	2.3 × 10^–^^4^ ± 3 × 10^–^^5^
*R*_adj_^2^	0.991	0.957
SD, mg g^–^^1^	0.78	2.94
BIC	–5.3	44.7
Mixed 1,2-order rate equation model		
*q*_e,cal_, mg g^–^^1^	36.7 ± 0.9	68 ± 3
*k*_1_, min^–1^	0.002 ± 0.001	2 × 10^–^^4^ ± 1 × 10^–^^4^
*f*_2_	0.93 ± 0.06	0.987 ± 0.009
*R*_adj_^2^	0.990	0.954
SD, mg g^–^^1^	0.80	2.94
BIC	–2.9	47.7
Intraparticle diffusion model		
*K*_D_, mg g^–^^1^ min^–^^0.5^	1.3 ± 0.2	1.9 ± 0.1
*C*, mg g^–^^1^	14 ± 2	21 ± 1
*R*_adj_^2^	0.900	0.990

Notably, the native lignin did not effectively adsorb 2,4-D, and removal efficiency was within experimental errors. This demonstrates that the one-step pyrolysis is an effective route for the valorization of kraft lignin as an effective sorbent characterized by high adsorption capacity at the pH region typical for groundwater and agricultural waste.

#### Kinetics

The influence of phase contact time on the adsorption process was carried out for two concentrations of 2,4-D which represent both low- and high-concertation regions in the equilibrium study. The experimental kinetics data and the fitting plots obtained by PFO, PSO, and MOE kinetic models are shown in Figure 6c. The fitting parameters are summarized in Table 1. Analysis of statistical criteria showed that for both concentrations, the PFO model resulted in worse SD and *R*_adj_^2^ values compared to PSO and MOE models. The differences between BIC parameters were also higher than 10 in couples PFO/PSO and PFO/MOE. The obtained results indicate that PFO is not a suitable model for the fitting of 2,4-D kinetics. Comparing the PSO and MOE models showed that the former model provided a better fitting of kinetics. Another indicator for the selection of PSO as the best option is the nearness of parameter *f*_2_ to unity (0.93 and 0.987 for low and high concentrations of 2,4-D, respectively). All obtained results indicate that herbicide adsorption onto N-AC is dominated by a PSO process. The initial sorption rates *h*_0_ (mg g^–1^ min^–1^) were estimated according to the equation proposed by Ho^68^ and using the fitted parameters of the PSO model
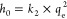
14where *k*_2_ is the rate constant [min^–1^ (g mg^–1^)], *q*_e_ is the adsorbed amount of 2,4-D at equilibrium (mg g^–1^), and 2 is the order of the kinetic model. The calculated values were 1.09 ± 0.01 and 1.08 ± 0.01 mg g^–1^ min^–1^ for 30 and 100 mg L^–1^ 2,4-D, respectively.

Plotting the adsorption curves as *q*_t_ vs *t*^0.5^ showed three linear regions (Figure 6d), indicating that the adsorption process involves three kinetic stages (or adsorption rates).^69^ The first linear range is a fast adsorption process and is referred to the film diffusion (transport of 2,4-D species through water covering N-AC particles) and followed by the adsorption of herbicide on the external surface of N-AC. The second range is attributed to intraparticle diffusion, the transport of 2,4-D species to the pores of N-AC. It is a rate-limiting step of the adsorption process and can be described by Weber–Morris intraparticle diffusion model (eq 7). The calculated values of *K*_D_ were 1.3 ± 0.2 and 1.9 ± 0.1 mg g^–1^ min^–0.5^ for low and high concentrations of 2,4-D. The last range corresponds to the diffusion of the 2,4-D species into micropores of N-AC, from which the system reaches equilibrium.

#### Adsorption of 2,4-D from Simulated and Actual Natural Water Samples

**Figure 7:**
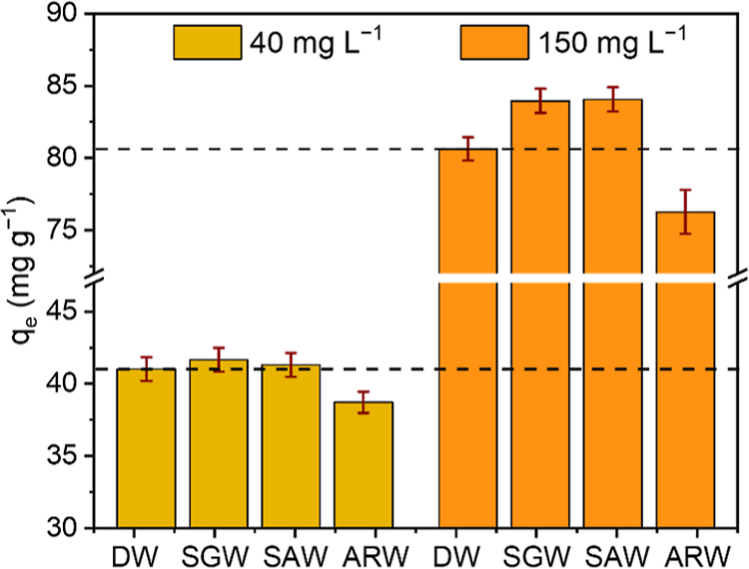
Adsorption value of 2,4-D from simulated and actual water samples containing 40 and 150 mg L^–1^ herbicide. Note: DW—distilled water; SGW—simulated groundwater; SAW—simulated agricultural water; and ARW—actual river water.

As the adsorption experiment proved that N-AC was an efficient adsorbent for removing 2,4-D from distilled water solutions (DW), the material was subjected to the treatment of SGW sample, SAW sample from tomato greenhouse, and actual water sample from Fyris river in Uppsala, Sweden (ARW). The comparison of adsorption capacity of N-AC toward 2,4-D from DW, SGW, SAW, and ARW with addition of the herbicide to reach its initial concentration on the level of 40 and 150 mg L^–1^ is presented in Figure 7. According to the results, adsorption of 2,4-D from simulated water solutions slightly increased compared to DW: with application of lower concentration of 2,4-D (40 mg L^–1^), the declination did not exceed the experimental uncertainties (41.0 ± 0.8, 41.7 ± 0.8, and 41.3 ± 0.8 mg g^–1^ for DW, SGW, and SAW correspondingly), while for 150 mg L^–1^ 2,4-D, it was 4–5% (80.6 ± 0.8, 83.9 ± 0.8, and 84.1 ± 0.8 mg g^–1^ for DW, SGW, and SAW, correspondingly). This increasing is explained by the drift of pH in DW to a more basic value due to the high pH_pzc_, when SGW and SAW contain components with buffer behavior. After the complete process in ARW, the adsorption values diminished to 38.7 ± 0.7 and 76 ± 1 mg g^–1^ for low and high initial concentrations of 2,4-D correspondingly. This minor decrease could be caused by the competition with other organic species-natural organic matter presented in ARW. Therefore, the adsorption in this medium and simulated samples is very high, proving efficiency of N-AC for treating even actual water objects rich in organic–inorganic contaminants.

## Conclusions

The study presented herein focuses on converting kraft lignin into a cost-effective and practical porous adsorbent for removing toxic and unwanted pollutants from air and aqueous environments. This involved a one-step process where lignin was carbonized into N-AC using nitric acid as a nonmetal activation agent and urea as an affordable nitrogen dopant and activation agent. The resulting material, denoted as N-AC, underwent comprehensive characterization, and its adsorption capacity was assessed for the removal of CO_2_ and herbicide 2,4-D.

N-AC exhibited significant microporosity, with pore diameters of 0.4 and 0.6 nm resulting in a remarkable specific surface area of 1000 m^2^ g^–1^. Further analysis, including Boehm’s titration, the pH_zpc_ test, FTIR, and XPS, indicated a substantial nitrogen content that influenced N-AC’s adsorption performance. Specifically, N-AC displayed effective CO_2_ capture and exceptional selectivity at a partial pressure of 0.15 bar. Notably, a single gram of N-AC was capable of adsorbing 1.4 mmol of CO_2_ with a selectivity over N_2_ of 27.3 at 20 °C and 1.2 mmol of CO_2_ with a selectivity over N_2_ of 17.8 at 25 °C (both values estimated for a total pressure of 1 bar). The presence of surface cationic groups significantly enhanced N-AC’s adsorption capacity for anionic organic contaminants under neutral conditions, such as 2,4-D. The adsorption of 2,4-D followed PSO kinetics with an estimated initial sorption rate of 1.085 ± 0.005 mg g^–1^ min^–1^. The Langmuir–Freundlich model provided the best fit for the experimental adsorption data, estimating N-AC adsorption capacity at 96 ± 6 mg g^–1^, while the experimental value was determined to be 85 ± 2 mg g^–1^ (with a removal efficiency of 28%). Notably, N-AC maintained its high adsorption capacity for 2,4-D when applied to SGW and wastewater from a tomato greenhouse. Even in the case of an actual river sample from the Fyris River in Uppsala, Sweden, N-AC performance experienced only a modest decrease of 4–5%.

## Supporting Information Available

Experimental procedure for kraft lignin characterization; composition of agricultural water from tomato greenhouses; functional group content of kraft lignin and N-AC; TGA of kraft lignin; XRD data of N-AC; additional data of N_2_ and CO_2_ adsorption on N-AC; and comparison analysis of cost production, technological aspects, and performance (CO_2_ capacity and selectivity) of N-AC and reported biomass-derived porous nitrogen-doped CMs (PDF)
