# Supplementary material for: Kraft Lignin-Derived Microporous Nitrogen-Doped Carbon Adsorbent for Air and Water Purification
Source: ACS Appl Mater Interfaces. 2024 Jan 9;16(3):3427–41. doi: 10.1021/acsami.3c15659 (PMC10811628; doi:10.1021/acsami.3c15659)
Supplement: Supplementary file 1 — am3c15659_si_001.pdf [file am3c15659_si_001.pdf]

Supporting Information

Kraft lignin-derived microporous nitrogen-doped carbon adsorbent for air and water purification

Oleg Tkachenko[1], Alina Nikolaichuk[1], Nataliia Fihurka[1], Andreas Backhaus[2], Julie B. Zimmerman[2], Maria Strømme[1], Tetyana M. Budnyak[1,2]*

[1]*Division of Nanotechnology and Functional Materials, Department of Materials Science and Engineering, The Ångström Laboratory, Uppsala University, Lägerhyddsvägen 1, Uppsala, 751 03, Sweden;*
[2]*Center for Green Chemistry and Green Engineering, School of the Environment, Yale University, 195 Prospect Street, New Haven, Connecticut 06511, United States.*

Corresponding Authors: Tetyana.Budnyak@angstrom.uu.se (TMB)

Number of Pages: 8

Number of Figures: 5

Number of Tables: 6

Experimental part

Molecular weight analysis of initial kraft lignin. The molecular weight of acetylated lignin, prepared according to the procedure[1], was analyzed using a Hitachi HPLC Chromaster system equipped with two LC Phenogel columns 50 Å and 500 Å (5 μm, 7.8×300 mm each and flow rate 1 mL min^{-1}) connected in series and with a UV detector (280 nm). The calibration was performed using ReadyCal-Kit polystyrene (266, 682, 1250, 2280, 3470, 4920, 9130, 15700, 21500, 28000, 44200, 66000 g mol^{-1}). The values of Mn and Mw were calculated using Clarity Chromatography Software.

^{31}P NMR analysis of initial kraft lignin. Quantification of lignin functional group content was performed using the procedure described by Argyropoulos *et. al.*[2] 31P NMR spectrum of phosphitylated sample was recorded using a 400MHz JEOL solution NMR spectrometer with the relaxation delay 10s and 128 scans.

Thermal Gravimetric Analysis of initial kraft lignin was carried out on a TGA/DSC/IR (Mettler Toledo, Columbus, OH, USA) instrument under the following operational conditions: a heating rate of 10°C min^{-1}, a dynamic atmosphere of air or nitrogen (50 mL·min^{-1}), a temperature range of 30–900 °C, and a sample mass of 2.5 mg.

Simulated agricultural wastewater. The composition of agricultural water from tomato greenhouses was provided by the Centre of Expertise Water Technology, Leeuwarden, Netherlands. The content is presented in Table S1.

Table S1. Inorganic composition of water from tomato greenhouses

Total nitrogen, mg L^{-1}	Total phosphorus, mg L^{-1}	Chemical Oxygen Demand, mg L^{-1}	NH_4 – N, mg L^{-1}	pH
198 ± 2	89 ± 1	117 ± 1	1.4 ± 0.1	6.2 ± 0.1

Results.

Lignin characterization

Table S2. Characterization results of initial kraft lignin

	Molecular weight of initial lignin, g mol^{-1}	
M_n	M_w	Polydispersity
1200	2800	2.38
	Functional groups content in initial lignin, mmol g^{-1}	
Aliphatic hydroxyl groups	Phenolic hydroxyl groups	Carboxyl groups
1.49	2.82	0.35

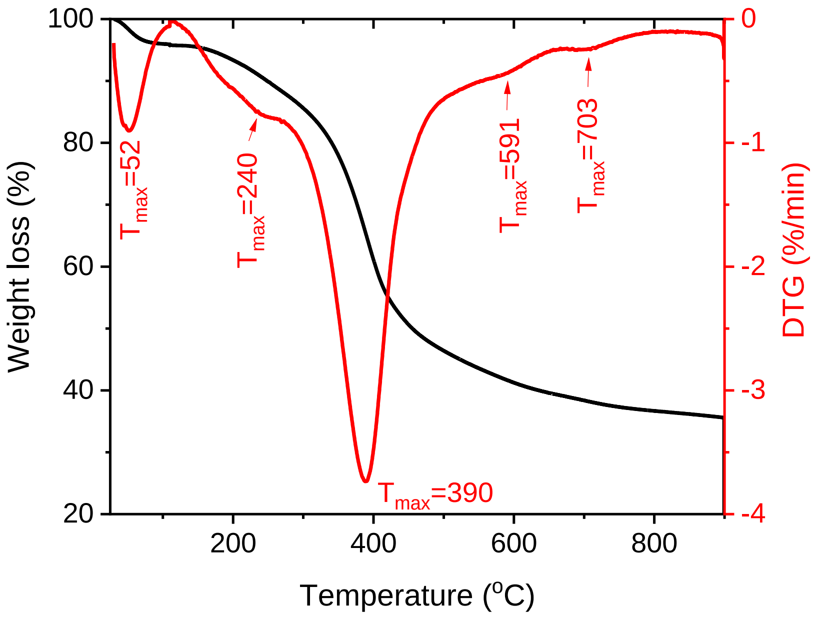

Figure S1. TG and DTG curves of kraft lignin thermal decomposition under N_2

The thermal stability of kraft lignin was investigated via TGA to evaluate the possible effective temperature range for carbonization. The thermogravimetric and differential thermogravimetric curves in Figure S1 represent the destruction processes of the used lignin in nitrogen atmosphere. The main characteristics of thermal decomposition are well described by Budnyak et. al.[3] As could be seen the plateau became visible after the temperature in the reactive camera reached 700 °C referring to the carbonization process. It should be noted the total weight loss changed from 61.6 % at 700 °C to 64.6 % at 900 °C, so this range was considered the optimal for carbonization of lignin.

Optimization of carbonization parameters

To better represent the impact of different carbonization temperatures and demonstrate that 800 °C is the most optimal temperature for the target purposes, the material adsorption behavior toward CO_2 and N_2 was compared at 20 °C and 25 °C, two of the most ambient temperatures. The presented isotherms (Figure S2) show that N-AC(800) demonstrated the highest adsorption capacities towards CO_2 for both temperatures. To quantify the preferences of 800 °C, adsorption capacities at 1 bar were collected in Table S3. As can be seen, the CO_2/N_2 capacity ratio for N-AC(800) was 11.2 at 20 °C 7.0 at 25 °C, which were higher than the similar values for other materials. In addition, the CO_2/N_2 selectivity of all three materials for the gaseous mixture (15 % CO_2 and 85 % N_2) was calculated using equation 1 and single adsorption isotherms. The found values (Table S3) were higher for N-AC(800 In summary, the N-AC(800) has better adsorption behavior than N-AC(700) and N-AC(900), and 800 °C is the most optimal temperature for the proposed method.

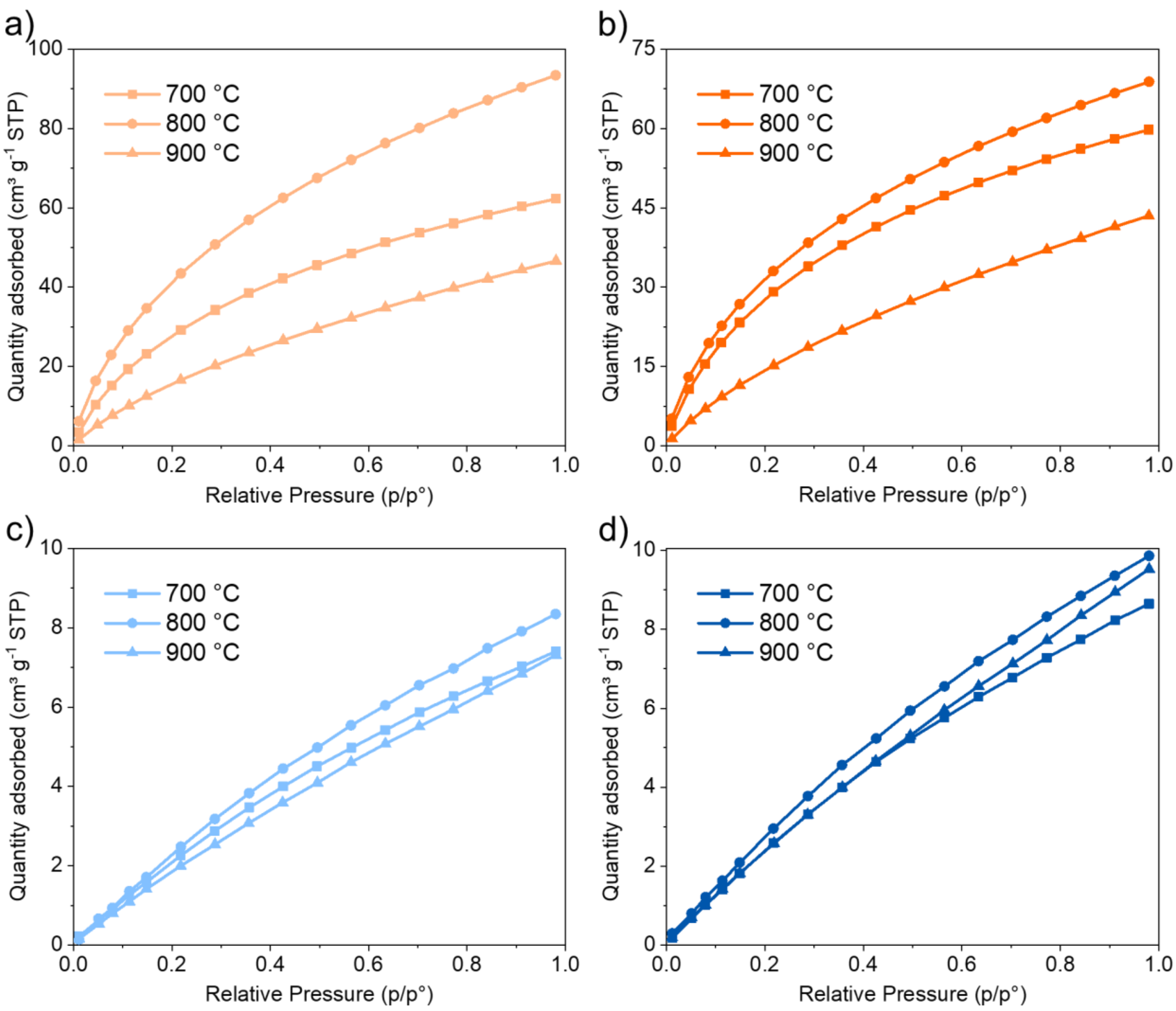

Figure S2. Adsorption isotherms of CO_2 (a and b) and N_2 (c and d) at 20 °C (a and c) and 25 °C (b and d) onto N-AC materials obtained at 700 °C, 800 °C and 900 °C.

Table S3. Comparison of the CO_2 and N_2 adsorption on N-AC obtained at three different carbonization temperatures

Adsorption of liquid N_2 at −196 °C						
Carbonization temperature, °C	Quantity of N_2 adsorbed at different pressure, mmol g^{-1}					
	0.05 p/p^0		0.16 p/p^0		0.98 p/p^0	
700	5.8		5.9		6.2	
800	12.0		12.5		12.8	
900	13.0		14.1		16.6	
Adsorption of CO_2 and N_2 at 20 °C and 25 °C						
Carbonization temperature, °C	CO_2		N_2			
	20 °C	25 °C	20 °C	25 °C	20 °C	25 °C
	Capacity at 1 bar, cm^3 g^{-1} STP				CO_2/N_2 capacity ratio	
700	62.3	59.8	7.4	8.6	8.4	6.9
800	93.4	68.9	8.3	9.9	11.2	7.0
900	46.6	43.5	7.3	9.5	6.4	4.6
	Capacity at 0.15 bar		Capacity at 0.85 bar		CO_2/N_2 selectivity	
700	23.1	23.3	6.7	7.7	19.7	17
800	34.7	26.7	7.5	8.8	26.7	17.1
900	12.5	11.4	6.4	8.4	11.1	7.8

XRD of N-AC

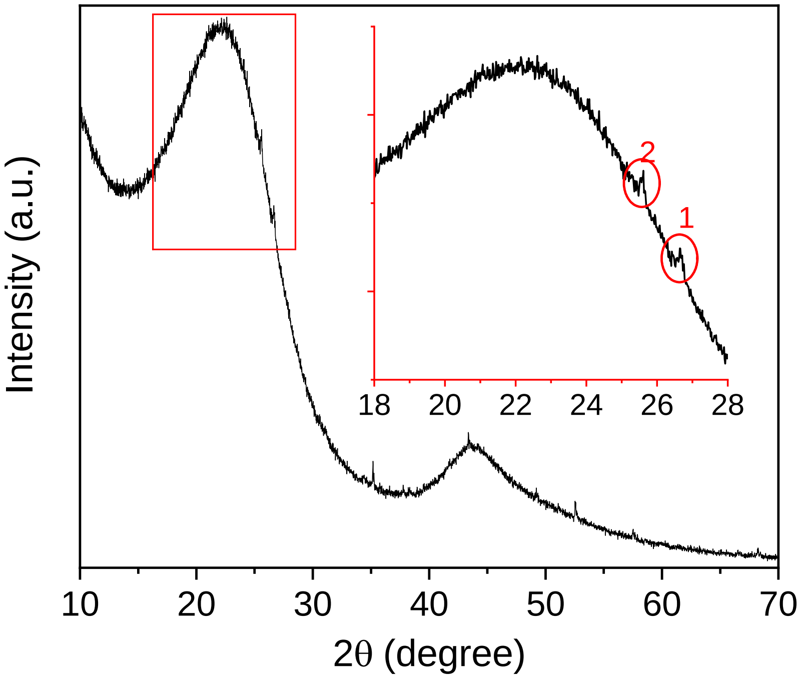

Figure S3. XRD of N-AC

Comparison analysis of N-AC production and gas adsorption performance

Table S4. Comparison of the CO_2 capacity and selectivity over N_2 at 25 °C of the reported biomass-derived porous nitrogen doped carbon materials

Origin	Activation agent (mass ratio)	Nitrogen agent (mass ratio)	Carbonization temperature, °C	N (wt %)	CO_2 capture, mmol g^{-1}		Selectivity (1 bar total pressure)	Ref.
					0.15 bar	1 bar		
Lignosulphonate	KOH (1:1)	Chitosan (1:3)	600	4.92[a]	0.9[c]	3.2	12[d]	7
De-alkaline lignin	KOH (1:1)	NH_3 (2.25L)	800/800	7.1[a]	1.4[c]	5.5	19.8[d]	8
Biomass waste hazelnut shell	KOH (1:2)	Melamine (1:1)	500/500/550	4.3[b]	1.2[c]	4.2	17[d]	9
Coconut shell	KOH (1:3)	Urea (1:1)	500/350/650	0.91[b]	1.2[c]	5.0	15[e]	10
Lignosulfonate acid sodium salt	KOH (1:3)	Urea (1:8)	200/700/800	1.17[a]	-	2.7	-	11
Organosolv lignin	KOH (1:4)	Adenine (1:1)	300/700	5.6[a]	-	4.8	17.9[e]	12
Biomass waste hazelnut shell	KOH (1:3)	Thiourea (1:1)	500/500/550	3.01[b]	1.2[c]	4.3	13[d] 17[e]	13
Dealkalized lignin	KOH (1:2)	No information	450/600	0.79[b]	-	3.9	17[e]	14
Black liquor lignin	KOH (1:2)	No agent	800	---	0.9[c]	3.6	12.7	15
Kraft lignin	HNO_3 (1:1.25)	Urea (1:0.3)	800	4.2[a] 3.5[b]	1.2	3.4	17.8[f]	This work

[a] N-content based on XPS analysis;
[b] N-content based on elemental analysis;
[c] the data were recovered from single-component adsorption isotherm;
[d] calculated using eq. 1 and data from single-component adsorption isotherm;
[e] the Authors calculated the value for 10/90 mixture of CO_2/N_2;
[f] the value was predicted using IAST model.

Table S5. Comparison of consumable, reagent cost, yield, and time for preparation of the reported biomass-derived porous nitrogen-doped carbon materials

Ref.	Yield, %	Mass ratio of carbon source/KOH	m(KOH), kg	Cost per unit, USD	KOH agent cost, USD	N-Agent	Mass ratio of carbon source /N-Agent	m(N-Agent), kg	Cost per unit, USD	N-Agent cost, USD	Total cost, USD	Time[a]
The cost of preparation[b] 1 kg N-containing carbon from biowaste												
7	20[c]	1:1	5		287.9	Chitosan	3	15	274.6 for 0.25kg	16473.0	16760.9	3h 56min
9	20[c]	1:2	10		267.9	Melamine	1	5	114.0 for 3 kg	190.0	457.9	9h 58min
10	20[c]	1:3	15		401.9	Urea	1	5	178.6 for 5 kg	178.6	580.5	8h 42 min
11	20[c]	1:3	15	287.8 for 5 kg	401.9	Urea	8	40	178.6 for 5 kg	1428.8	1830.7	19h 52min
12	20[c]	1:4	20		535.8	Adenine	1	5	2930.8 for 1kg	14653.8	15189.6	13h 46min
13	30.4[d]	1:3	9.9		264.4	Thiourea	1	3.3	327.8 for 5 kg	215.6	480.0	9h 58 min
14	20[c]	1:2	10		267.9	unknown	---	---	---	---	267.9	7h 22min
15	21.1	1:2	9.5		253.9	----	---	---	---	---	253.9	3h 36min
This work	30.0	1:1.25[e]	4.2[e]	134.0 for 2.5L (65%)	246.9[e]	Urea	0.3	1	178.6 for 5 kg	35.7	282.6	3h 36min
The cost of preparation[b] 5 kg N-containing carbon from biowaste												
7	20[c]	1:1	25		1439.3	Chitosan	3	75	274.6 for 0.25kg	82365.0	83804.3	3h 56min
9	20[c]	1:2	50		1339.5	Melamine	1	25	114.0 for 3 kg	950.0	2289.5	9h 58min
10	20[c]	1:3	75		2009.3	Urea	1	25	178.6 for 5 kg	893.0	2902.3	8h 42 min
11	20[c]	1:3	75	287.8 for 5 kg	2009.3	Urea	8	200	178.6 for 5 kg	7144.0	9153.3	19h 52min
12	20[c]	1:4	100		2679.0	Adenine	1	25	2930.8 for 1kg	73268.8	75947.8	13h 46min
13	30.4[d]	1:3	75		1321.9	Thiourea	1	16.5	327.8 for 5 kg	1078.1	2400.0	9h 58 min
14	20[c]	1:2	50		1339.5	unknown	---	---	---	---	1339.5	7h 22min
15	21.1	1:2	50		1269.7	----	---	---	---	---	1269.7	3h 36min
This work	30.0	1:1.25[e]	20.8[e]	545.3 for 25L (65%)	502.5[e]	Urea	0.3	5	178.6 for 5 kg	178.6	681.1	3h 36min

[a] Time required for long and high-temperature treatment during the carbonization process;

[b] The calculation covered only spences for the activator and N-modified agents since carbon sources were considered as biowaste. The information about the reagent cost was taken from the Sigma Aldrich website for Sweden;

[c] assumption while the authors did no provide information about the yield of the N-containing carbon;

[d] information provided for two steps, while one step is missed;

[e] nitric acid was used instead of KOH.

Simulation of gas adsorption

The single-site Langmuir adsorption model assumes the formation of a monolayer of gas molecules onto energy-identical and homogeneous sites (*N*, mmol g^{-1}) of the adsorbent, the affinity of which is described by parameter *K* (bar^{-1}):

$$n_i^o = N_{max} \frac{K \times P_i}{1 + K \times P_i} \tag{S1}$$

where n_i^o is adsorbed quantity at total pressure P_i (bar^{-1}).

Henry's law (model) is:

$$n_i^o(P) = K_H P \tag{S2}$$

where K_H is the Henry coefficient (units: loading/pressure).

Ideal adsorbed solution theory (IAST) proposed by Myers and Prausnitz[4] is useful in predicting multi-adsorption isotherms. The construction of these isotherms assumes an ideal mixture of the gases using the pure-component adsorption isotherm. The temperature is fixed for both pure-component and simulated multi-mixed isotherms. The IAST calculation for CO_2/N_2 mixture is based on the following equations[5]:

$$P_{y_{CO_2}} = P_{CO_2}^o \times \chi_{CO_2} \qquad \text{(S3),}$$

$$\chi_{CO_2} = \frac{P_{N_2}^o - P}{P_{N_2}^o - P_{CO_2}^o} \qquad \text{(S4),}$$

$$\frac{1}{N_{tot}} = \frac{\chi_{CO_2}}{n_{CO_2}(P_{CO_2}^o)} + \frac{1 - \chi_{CO_2}}{n_{N_2}(P_{N_2}^o)} \qquad \text{(S5),}$$

$$\int_0^{P_{CO_2}^o} \frac{n_{CO_2}(p)}{p} dp = \int_0^{P_{N_2}^o} \frac{n_{N_2}(p)}{p} dp \qquad \text{(S6),}$$

where P is the total pressure (bar), y_{CO_2} is the molar fraction of carbon dioxide, χ_{CO_2} is the molar fraction of CO_2 in the adsorbed phase, n_{CO_2} and n_{N_2} are equilibrium adsorption (mmol g^{-1}) at pressure *P*, $P_{CO_2}^o$ and $P_{N_2}^o$ are pure component hypothetical pressure which yields the same spreading pressure as that of the mixture (bar), N_{tot} the total gas adsorption (mmol g^{-1}). To solve these equations, Python package pyIAST[6] was used.

Table S6. Fitting parameters for the single-components CO_2 and N_2 adsorptions on N-AC

Gas	*Temperature,* °C	*Model*	N_{max}, mmol g^{-1}	*K*, bar^{-1}	RMSE
CO_2	20	Langmuir	5.85	2.25	7.6 10^{-2}
N_2		Henry	-	0.405	1.7 10^{-2}
CO_2	25	Langmuir	4.15	2.53	7.6 10^{-2}
N_2		Henry	-	0.480	2.1 10^{-2}

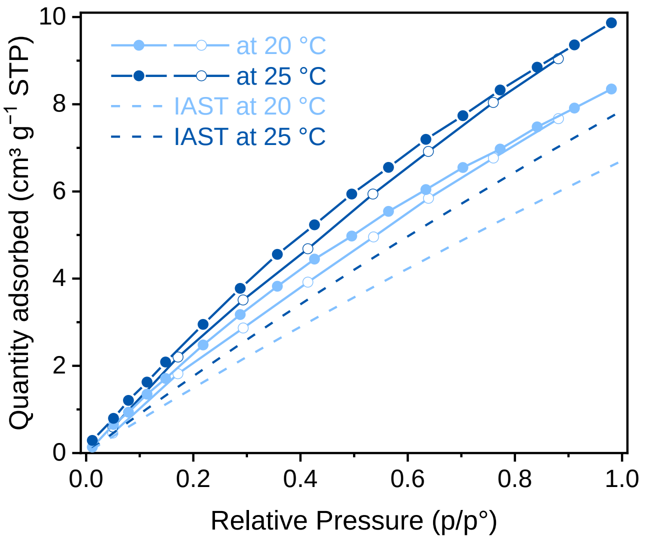

Figure S4. Adsorption (filled symbols) and desorption (hollow symbols) isotherms of N_2 at 20 °C and 25 °C (solid lines) and simulated adsorption IAST isotherms of N_2 (dash lines) for gaseous mixture (15 % of CO_2 and 85 % of N_2)

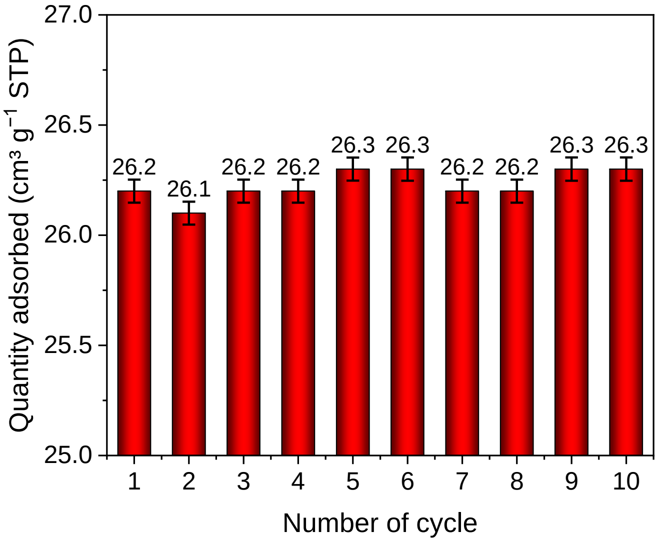

Figure S5. CO_2 adsorption capacity of N-AC at 25 °C and 0.15 bar during ten consequently adsorption-desorption cycles.

REFERENCES

(1) Kong, F.; Wang, S.; Gao, W.; Fatehi, P. Novel Pathway to Produce High Molecular Weight Kraft Lignin–Acrylic Acid Polymers in Acidic Suspension Systems. *RSC Adv* **2018**, *8* (22), 12322–12336. https://doi.org/10.1039/C7RA12971H.

(2) Argyropoulos, D. S.; Pajer, N.; Crestini, C. Quantitative 31P NMR Analysis of Lignins and Tannins. *Journal of Visualized Experiments* **2021**, No. 174. https://doi.org/10.3791/62696.

(3) Budnyak, T.; Aminzadeh, S.; Pylypchuk, I.; Riazanova, A.; Tertykh, V.; Lindström, M.; Sevastyanova, O. Peculiarities of Synthesis and Properties of Lignin–Silica Nanocomposites Prepared by Sol-Gel Method. *Nanomaterials* **2018**, *8* (11), 950. https://doi.org/10.3390/nano8110950.

(4) Myers, A. L.; Prausnitz, J. M. Thermodynamics of Mixed-Gas Adsorption. *AIChE Journal* **1965**, *11* (1), 121–127. https://doi.org/10.1002/aic.690110125.

(5) Åhlén, M.; Kapaca, E.; Hedbom, D.; Willhammar, T.; Strømme, M.; Cheung, O. Gas Sorption Properties and Kinetics of Porous Bismuth-Based Metal-Organic Frameworks and the Selective CO2 and SF6 Sorption on a New Bismuth Trimesate-Based Structure UU-200. *Microporous and Mesoporous Materials* **2022**, *329*, 111548. https://doi.org/10.1016/j.micromeso.2021.111548.

(6) Simon, C. M.; Smit, B.; Haranczyk, M. PyIAST: Ideal Adsorbed Solution Theory (IAST) Python Package. *Comput Phys Commun* **2016**, *200*, 364–380. https://doi.org/10.1016/j.cpc.2015.11.016.

(7) Shao, L.; Wan, H.; Wang, L.; Wang, J.; Liu, Z.; Wu, Z.; Zhan, P.; Zhang, L.; Ma, X.; Huang, J. N-Doped Highly Microporous Carbon Derived from the Self-Assembled Lignin/Chitosan Composites Beads for Selective CO2 Capture and Efficient p-Nitrophenol Adsorption. *Sep Purif Technol* **2023**, *313*, 123440. https://doi.org/10.1016/j.seppur.2023.123440.

(8) Saha, D.; Van Bramer, S. E.; Orkoulas, G.; Ho, H.-C.; Chen, J.; Henley, D. K. CO2 Capture in Lignin-Derived and Nitrogen-Doped Hierarchical Porous Carbons. *Carbon N Y* **2017**, *121*, 257–266. https://doi.org/10.1016/j.carbon.2017.05.088.

(9) Pang, R.; Lu, T.; Shao, J.; Wang, L.; Wu, X.; Qian, X.; Hu, X. Highly Efficient Nitrogen-Doped Porous Carbonaceous CO_2 Adsorbents Derived from Biomass. *Energy & Fuels* **2021**, *35* (2), 1620–1628. https://doi.org/10.1021/acs.energyfuels.0c03832.

(10) Chen, J.; Yang, J.; Hu, G.; Hu, X.; Li, Z.; Shen, S.; Radosz, M.; Fan, M. Enhanced CO_2 Capture Capacity of Nitrogen-Doped Biomass-Derived Porous Carbons. *ACS Sustain Chem Eng* **2016**, *4* (3), 1439–1445. https://doi.org/10.1021/acssuschemeng.5b01425.

(11) Park, S.; Choi, M. S.; Park, H. S. Nitrogen-Doped Nanoporous Carbons Derived from Lignin for High CO2 Capacity. *Carbon Letters* **2019**, *29* (3), 289–296. https://doi.org/10.1007/s42823-019-00025-z.

(12) Demir, M.; Tessema, T.-D.; Farghaly, A. A.; Nyankson, E.; Saraswat, S. K.; Aksoy, B.; Islamoglu, T.; Collinson, M. M.; El-Kaderi, H. M.; Gupta, R. B. Lignin-Derived Heteroatom-Doped Porous Carbons for Supercapacitor and CO_2 Capture Applications. *Int J Energy Res* **2018**, *42* (8), 2686–2700. https://doi.org/10.1002/er.4058.

(13) Ma, C.; Lu, T.; Shao, J.; Huang, J.; Hu, X.; Wang, L. Biomass Derived Nitrogen and Sulfur Co-Doped Porous Carbons for Efficient CO2 Adsorption. *Sep Purif Technol* **2022**, *281*, 119899. https://doi.org/10.1016/j.seppur.2021.119899.

(14) Gong, L.; Bao, A. High-Value Utilization of Lignin to Prepare N,O-Codoped Porous Carbon as a High-Performance Adsorbent for Carbon Dioxide Capture. *Journal of CO2 Utilization* **2023**, *68*, 102374. https://doi.org/10.1016/j.jcou.2022.102374.

(15) Zhao, J.; Zhang, W.; Shen, D.; Zhang, H.; Wang, Z. Preparation of Porous Carbon Materials from Black Liquor Lignin and Its Utilization as CO2 Adsorbents. *Journal of the Energy Institute* **2023**, *107*, 101179. https://doi.org/10.1016/j.joei.2023.101179.
